# *SUB1* Plays a Negative Role during Starvation Induced Sporulation Program in *Saccharomyces cerevisiae*

**DOI:** 10.1371/journal.pone.0132350

**Published:** 2015-07-06

**Authors:** Ritu Gupta, Parag P. Sadhale, Usha Vijayraghavan

**Affiliations:** Department of Microbiology and Cell Biology, Indian Institute of Science, Bangalore, 560012, India; California Department of Public Health, UNITED STATES

## Abstract

*Saccharomyces cerevisiae* Sub1 is involved in several cellular processes such as, transcription initiation, elongation, mRNA processing and DNA repair. It has also been reported to provide cellular resistance during conditions of oxidative DNA damage and osmotic stress. Here, we report a novel role of *SUB1* during starvation stress-induced sporulation, which leads to meiosis and spore formation in diploid yeast cells. Deletion of *SUB1* gene significantly increased sporulation efficiency as compared to the wild-type cells in S288c genetic background. Whereas, the sporulation functions of the *sub1*(Y66A) missense mutant were similar to Sub1. *SUB1* transcript and protein levels are downregulated during sporulation, in highly synchronized and sporulation proficient wild-type SK1 cells. The changes in Sub1 levels during sporulation cascade correlate with the induction of middle sporulation gene expression. Deletion of *SUB1* increased middle sporulation gene transcript levels with no effect on their induction kinetics. In wild-type cells, Sub1 associates with chromatin at these loci in a temporal pattern that correlates with their enhanced gene expression seen in *sub1Δ* cells. We show that *SUB1* genetically interacts with *HOS2*, which led us to speculate that Sub1 might function with Set3 repressor complex during sporulation. Positive Cofactor 4, human homolog of Sub1, complemented the *sub1Δ* sporulation phenotype, suggesting conservation of function. Taken together, our results suggest that *SUB1* acts as a negative regulator of sporulation.

## Introduction

All eukaryotic organisms ranging from unicellular yeast to multicellular humans possess a great deal of complexity. Cellular processes such as transcription, translation, splicing, DNA replication, chromatin organization, chromatin remodelling are carried out by specialized macromolecular complexes, which are aided by multiple accessory factors. These factors function in a highly coordinated manner, both under normal growth conditions and during various stress response. Many of the factors have multi-facet roles where they are known to be involved in different processes at the same time, thereby helping in coordinating these functions and also indirectly helping in energy conservation for the cell by bypasing the need to synthesize individual factors for each process. Sub1 is one such protein, which is known for its pleiotropic cellular activities. Sub1 originally identified as a *Su*ppressor of TFII*B* mutations [1] and as Transcriptional Stimulatory Protein 1 [2], was first ascribed functions in transcriptional control of gene expression. It is a highly conserved protein present from yeast to humans [1,2]. It plays important roles during transcription by modulating the association of RNA Pol II throughout several constitutively transcribed genes [3–6]. These effects are likely direct as Sub1 has been reported to bind to the promoter region of almost all the constitutively—transcribed RNA Pol II [4,5] and Pol III genes [4] and also throughout the transcribed region of genes [3,7]. More recently, it was identified as a component of preinitiation complex by *in vitro* studies and found to show strong genetic interactions with TFIIE and TFIIH factors [5].

Starvation is a universal condition faced by all organisms and survival through prolonged starvation could hold evolutionary significance. The effects of mild starvation or calorie restriction on extension of life span are universal as well [8]; hence many regulators of starvation processes are likely to be similar even if the actual response to starvation may vary among different organisms. Study of starvation response using the yeast *S*. *cerevisiae* as a model has been insightful and indeed effect of mild starvation /calorie restriction on increased lifespan has been shown long ago in yeast [9]. As a response to starvation in *S*. *cerevisiae*, a/α diploid cells can adopt two distinct developmental outcomes, one leading to meiosis and sporulation during severe starvation [10], while the other leading to pseudohyphal morphogenesis under conditions of somewhat milder starvation [11]. Sporulation process is mainly governed by the nutritional status and mating type of the cell with absence of nitrogen and presence of a nonfermentable carbon source such as acetate being the key triggers for sporulation [10,12,13]. Expression of both *MATa* and *MATα* loci are necessary for sporulation, thereby, rendering the haploids and a/a and α/α diploid cells unable to sporulate [10,14]. Sporulation process once committed goes through a temporal cascade of transcriptional regulation of genes, which are categorized as- early, middle, mid-late and late genes based on the time kinetics of their onset [15,16]. Out of 6,200 genes in yeast genome, so far 1,600 genes have been shown to be involved during sporulation in SK1 and W303 strain backgrounds [16]. Analysis of gene deletion strains in genome-wide screen in S288c strain background has identified additional 200 genes to be positive regulators and 100 genes to be negative regulators of sporulation [17]. This study indicated Sub1 to be one among the negative regulators of sporulation. The number of genes known to negatively regulate sporulation is far lower as compared to those that are activators of sporulation. While negative regulators are categorized into different classes based on their function, for example, transcription, mitosis, cell cycle control and pseudohyphal differentiation [17], their mechanism of action is poorly studied.

Here, we present a detailed study on Sub1 to uncover its mechanism of action as a negative regulator of sporulation. Our data show deletion of *SUB1* significantly increases sporulation efficiency when compared with wild-type S288c cells. Additionally, we find that human PC4 can complement the sporulation phenotype of *sub1Δ/sub1Δ* S288c deletion mutant, suggesting it is evolutionarily conserved. Moreover, our findings in SK1 strain background, suggest that Sub1 regulates the expression levels of middle sporulation genes. These transcriptional changes are mediated by direct recruitment of Sub1 to the promoters of these genes.

## Materials and Methods

### Yeast strains used in this study

All the strains used in this study are in S288c and SK1 genetic backgrounds (listed in Table 1). Wild-type and *sub1Δ* haploid yeast strains of S288c background were purchased from Euroscarf. *sub1Δ/sub1Δ* (2n) was generated by crossing haploid *sub1Δ* allele of opposite mating type. Wild-type diploid in SK1 background (NKY3822) was obtained from Nancy Kleckner’s lab.

**Table 1:** Yeast strains used in this study.

Strain	Genotype	Source
WT (BY4741)	*MATa his3Δ1 leu2Δ0 met15Δ0 ura3Δ0*	Euroscarf
WT (BY4743)	*MATa/MATα his3Δ1/his3Δ1 leu2Δ0/leu2Δ0 met15Δ0/MET15 LYS2/lys2Δ0 ura3Δ0/ura3Δ0*	Euroscarf
*sub1Δ*::*kanMX4* (BY4741)	*MATa his3Δ1 leu2Δ0 met15Δ0 ura3Δ0 sub1Δ*::*kanMX4*	Euroscarf
*sub1Δ*::*kanMX4* (BY4742)	*MATα his3Δ1 leu2Δ0 lys2Δ0 ura3Δ0 sub1Δ*::*kanMX4*	Euroscarf
*sub1Δ*::*kanMX4* (S288c)	*MATa/MATα his3Δ1/his3Δ1 leu2Δ0/leu2Δ 0 met15Δ0/MET15 LYS2/lys2Δ0 ura3Δ0/ura3Δ0 sub1Δ*::*kanMX4/sub1Δ*::*kanMX4*	This study
NKY3822 (SK1)	*MATa/MATα ho*::*hisG/ ho*::*hisG leu2*::*hisG/ leu2*::*hisG ura3 (Δsma-pst)/ ura3 (Δsma-pst)*	Nancy Kleckner
*sub1Δ*::*natMX4* (SK1)	*MATa/MATα ho*::*hisG/ ho*::*hisG leu2*::*hisG/ leu2*::*hisG ura3 (Δsma-pst)/ ura3 (Δsma-pst) sub1Δ*::*natMX4/ sub1Δ*::*natMX4*	This study
WT *SUB1*-TAP (SK1)	*MATa/MATα ho*::*hisG/ ho*::*hisG leu2*::*hisG/ leu2*::*hisG ura3 (Δsma-pst)/ ura3 (Δsma-pst) SUB1*::*TAP-KanMX4/ SUB1*::*TAP-KanMX4*	This study

*sub1Δ/sub1Δ* (2n) in SK1 background was generated by PCR—mediated disruption using *natMX4* from pAG25 plasmid [18]. Primers were designed to generate a PCR fragment carrying the *PAgTEF1-natMX4-TAgTEF1* cassette and 40 nucleotides from upstream and downstream of start and stop codon of *SUB1* (S1 Table). The strain was confirmed by PCR and Southern blot analysis.

*SUB1*-TAP (2n) strain was generated by chromosomal integration of PCR amplified *TAP-kanMX6* cassette from pYM13 plasmid [19] at the C-terminal end of *SUB1* ORFs. The strain was confirmed by PCR and Southern blot analysis.

### Plasmids used in this study

The plasmids used in this study are listed in Table 2. For overexpression studies in S288c background, wild-type *SUB1* ORF (1–879 nt) was PCR amplified using genomic S288c DNA as template. The amplified product was cloned as EcoRI-XhoI fragment in p426TEF (*2μm*, *URA3*) (pPS189) plasmid [20] to generate pRV765 plasmid.

**Table 2:** Plasmids used in this study.

Plasmid	Description	Source/reference
pAG25	*PAgTEF1-natMX4-TAgTEF1*	18
pYM13	*TAP-kanMX4*	19
pPS189	*PTEF2* in pRS426 *URA3*	20
pRV765	*SUB1* ORF in pPS189	This study
pRV920	*sub1*(Y66A) allele in pPS189	This study
pRV917	PC4 cDNA in pPS189	This study
pPS887	GFP under P*TEF2* in pRS416 *URA3*	This study
pRV888	*SUB1* ORF in pPS887	This study
pRV921	*sub1*(Y66A) allele in pPS887	This study
pRV918	PC4 cDNA in pPS887	This study
pPS883	*SUB1* ORF-*6*X*His-HA-Protein A* under P*GAL1* in pBG1805 *URA3*	Open Biosystems

*sub1*(Y66A) missense mutant was generated by PCR-mediated site-directed mutagenesis method by changing codon 66 from TAT (tyrosine) to GCT (alanine). The mutagenized PCR product was cloned as EcoRI-XhoI fragment in p426TEF plasmid to generate pRV920 plasmid.

Human PC4 coding sequence (1–384 nt) was PCR amplified from a PC4 full-length cDNA pCMV clone. The amplified product was cloned as HindIII-XhoI fragment in p426TEF plasmid to generate pRV917 plasmid.

For localization studies, same constructs were cloned in pRS416-TEF2-GFP (*CEN6*, *URA3*) [20] (pPS887) plasmid to generate pRV888, pRV921 and pRV918 plasmids, where GFP was N-terminally fused to *SUB1*, *sub1*(Y66A) and PC4 respectively.

For qRT-PCR analysis in overexpression strains, the plasmid pBG1805 (*2μm*, *URA3*) where *SUB1* ORF (1–879 nt) with C terminal *6XHis-HA-Protein A* tag under *pGAL1* expression control (Table 2) was purchased from Open Biosystems (pPS883).

### Growth media and conditions

Vegetative yeast cultures were grown in YEPD medium (1% yeast extract, 2% peptone, 2% dextrose). For selection of plasmid, cells were grown in Synthetic drop-out (SD) medium (10% drop-out media, 10% dextrose and auxotrophic amino acids). For synchronization of cells during sporulation, cells were grown in YEPA (1% yeast extract, 2% peptone, 1% potassium acetate) or PSP2 [21] medium. Sporulation was carried out in liquid sporulation medium (1% potassium acetate plus 1/4^th^ auxotrophic amino acids).

### Sporulation assay

Cells were grown overnight in either YEPD or Synthetic drop-out (SD) medium (for plasmid selection). Cells were sub-cultured into YEPA or PSP2 media for synchronization and grown to an optical density at 600 nm (OD_600_) of ~ 0.8–1.0. Cells were collected by centrifugation, washed twice in water and once in 1% potassium acetate and resuspended in equal volume of sporulation medium (1% potassium acetate with 1/4^th^ auxotrophic amino acids). Cells were collected at indicated time points for Western blot or reverse transcription-quantitative PCR analysis.

For overexpression analysis of *SUB1* from the *GAL1* promoter, cells were grown in 2% raffinose containing synthetic drop-out medium to an optical density at 600 nm (OD_600_) of ~ 1.0. Subsequently, 2% galactose was added to this media and the culture was kept for 6 hours to induce the expression of *SUB1* from *GAL1* promoter. Cells grown in 2% raffinose containing synthetic drop-out medium, in the absence of galactose were used as uninduced controls for this experiment. Cells were then collected, washed in water and resuspended in equal volume of sporulation medium (1% potassium acetate with 1/4^th^ auxotrophic amino acids) to induce sporulation.

Throughout the study, % sporulation was calculated by counting the number of mature refractile asci (two, three or four spore) divided by the total number of cells induced to sporulate. Cells were analyzed using differential interference contrast (DIC) microscopy.

For scoring completion of meiosis, at least 200 cells stained with DAPI (4’,6-diamidino-2-phenylindole) were counted for each sample. Cells with a single nucleus were counted as vegetative (cells which have not undergone any meiotic division) and cells with three or four nuclei were counted as tetrads (cells which have undergone both meiotic—I and II divisions). Cells were analyzed using fluorescence microscopy.

Spore viability was determined by the dissection of at least 20 tetrads of *sub1Δ/sub1Δ* (2n) strain. Dissected spores were allowed to germinate on YPD medium for 2 days.

### RNA preparation and reverse transcription—quantitative PCR analysis

10 ml of cells grown to an optical density at 600 nm (OD_600_) of approximately 1 was collected at indicated time points. RNA was prepared using hot phenol beating method. 6 μg of total RNA for each sample was used for DNase I (Roche) treatment and cDNA synthesis using Superscript III enzyme (invitrogen). cDNA quantification was done by real-time PCR on an ABI Prism 7900HT System using Kapa SYBR Fast qPCR master mix. For qPCR analysis, three independent PCR reactions were performed with at least two independent biological replicates. Statistical significance was determined using Student *T*-test. The genes where transcript levels showed at least 2-fold change and statistical significance of P < 0.05 in different biological replicates were considered to be affected. The primers used for all transcripts analyzed are listed in S1 Table.

### Protein preparation and Western blot analysis

For sporulation time-course experiment, cells were harvested at different time points during sporulation and protein was isolated by trichloroacetic acid (TCA) precipitation method. Protein samples were electrophoretically resolved on SDS-polyacrylamide gels and subjected to Western blot analysis. Rabbit polyclonal anti-Protein A antibody (P3775) (1:1,000) from Sigma was used to detect Sub1-TAP protein. Mouse monoclonal anti-beta Actin antibody (ab8224) (1:2,500) from Abcam was used for loading control. For GFP expression in mitotically growing cultures, rabbit polyclonal anti-GFP antibody (ab6556) (1:1,000) from Abcam was used. HRP conjugated goat anti-rabbit or anti-mouse (1:10,000) from Sigma were used as the secondary antibodies. Quantification of the protein bands was carried out using multi-gauge software.

### Immunofluorescence assay

Cells were harvested, fixed in 90% ethanol. The cells were collected and resuspended in 1X phosphate-buffered saline (PBS) and stained with 1μl of 1mg/ml DAPI (4’,6-diamidino-2-phenylindole) for 10 min by incubation in dark. The cells were washed twice with water and then placed on a glass slide and photographed.

### Chromatin immunoprecipitation (ChIP) and quantitative PCR (qPCR) analysis

300 ml of cells was grown in YEPA medium to an optical density at 600 nm (OD_600_) of approximately 0.8–1.0. The cells were pelleted down, washed twice with water, once with 1% potassium acetate and resuspended in three flasks each containing 100 ml of sporulation medium. Two flasks were kept for shaking at 30°C for 2 and 5 hours and 0^th^ hour flask was immediately proceeded for further steps. At each time point during sporulation time-course, cells were cross-linked with formaldehyde (final concentration of 1%) for 20 min and quenched with glycine (final concentration of 125 mM) for 5 min. Cells were lysed by resuspending in spheroplasting buffer in presence of lyticase enzyme (Sigma), followed by shearing of DNA by sonication. 50 μl of the lysate was saved as an input, remaining chromatin was divided equally into immunoprecipitation (IP) fraction and no antibody fraction. Immunoprecipitation was carried out by adding polyclonal anti-Protein A antibody (Sigma) and kept for overnight at 4°C followed by incubation with Protein A- sepharose beads (Sigma) for 6–8 hours at 4°C. Beads were then washed twice with extraction buffer, once with high salt extraction buffer, once with LiCl wash buffer and once with TE (Tris-EDTA). DNA-Protein complexes were eluted from beads in elution buffer I and II by incubation at 65°C for 15 min. Cross-links were reversed by incubating at 65°C overnight followed by proteinase-K (Sigma) treatment. DNA was purified and precipitated at -80°C overnight. DNA quantification was done by real-time PCR on an ABI Prism 7900HT System using Kapa SYBR Fast qPCR master mix. For qPCR analysis, each reaction was carried out in three independent PCR reactions. Three independent biological replicates were analyzed for each data point. Input and mock IP with no antibody samples were the samples used for normalization and calculations were done as previously described [22]. Fold-enrichment was calculated as the ratio of immuno-precipitated DNA detected for *SMK1*, *SPS2* or *NDT80* promoter regions to the immuno-precipitated DNA for the *ACT1* promoter region as previously described [23]. The primers used for ChIP-qPCR analysis are listed in S1 Table.

## Results

### Deletion of *SUB1* increases sporulation efficiency of cells

**Fig 1:**
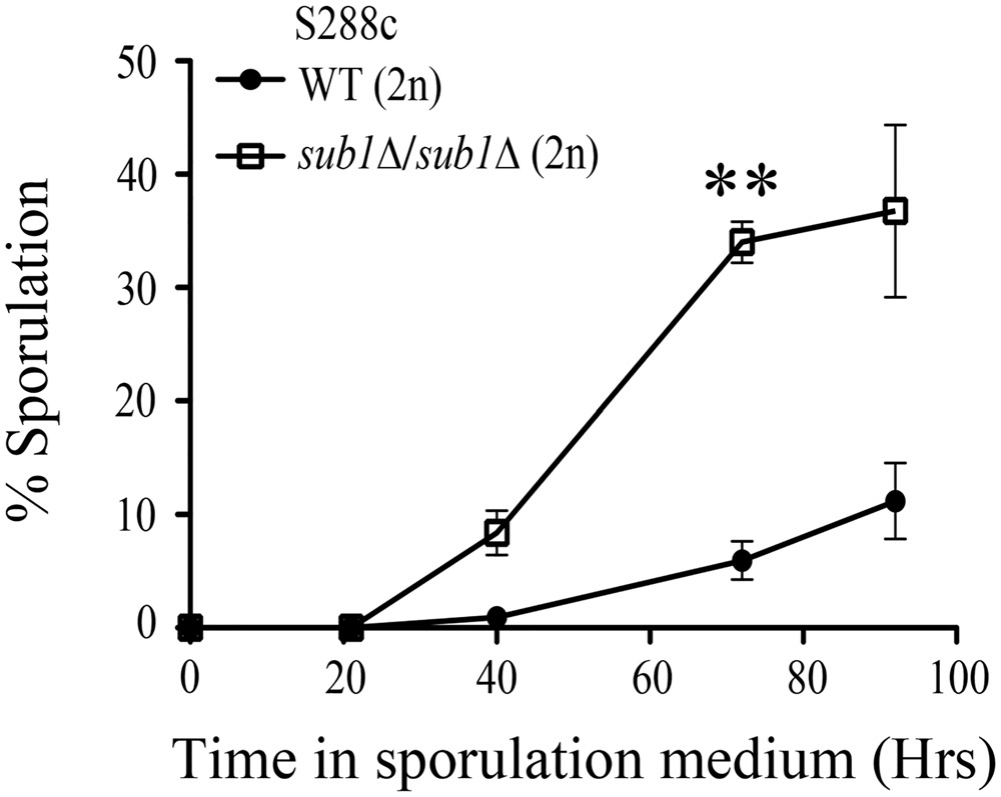
Deletion of *SUB1* shows an increase in sporulation efficiency compared to wild type cells. Sporulation was measured at different time points as indicated after transfer of cells from presporulation to sporulation medium. At least 1000 cells were counted at each time point in two independent replicate experiments. Double asterisk indicates P value of < 0.01.

In a previous whole-genome screen for mutants with altered sporulation efficiency in the *Saccharomyces cerevisiae* S288c strain, *SUB1* locus was identified as a negative regulator of sporulation [17]. Moreover, genome-wide gene expression analysis in the SK1 strain, with synchronized and highly efficient induction of sporulation, showed that *SUB1* transcript levels are reduced during sporulation [15,24]. Here, we analyzed the kinetics of asci formation in a diploid strain deleted for both the alleles of *SUB1* and compared to the isogenic wild-type strain. In the S288c genetic background, we generated homozygous diploid *sub1Δ* alleles by crossing haploid deletion strains obtained from Euroscarf. Sporulation of WT and *sub1Δ/sub1Δ* cells was induced by nitrogen starvation and use of non-fermentable sugar as the sole carbon source. Analysis of sporulation kinetics showed that deletion of *SUB1* resulted in an overall increase in sporulation efficiency, with nearly 5-fold increase in sporulation efficiency observed after 72 hours of transfer of *sub1Δ/sub1Δ* cells to sporulation medium as compared to that of the isogenic wild type diploid strain (Fig 1). Therefore, in agreement with the earlier genome-wide studies, we find that Sub1 plays a negative role in kinetics of sporulation in S288c strain background. We also examined the germination efficiency of haploid spores by dissecting 25 tetrads of *sub1Δ/sub1Δ* diploid cells (S1 Fig). Out of 100 spores dissected, 97 germinated on rich YPD medium. Therefore we conclude the viabilty of the haploid spores from *sub1Δ/sub1Δ* diploids is comparable to that from wild-type diploids.

### *sub1*(Y66A) missense mutant of Sub1 is competent for its sporulation function

**Fig 2:**
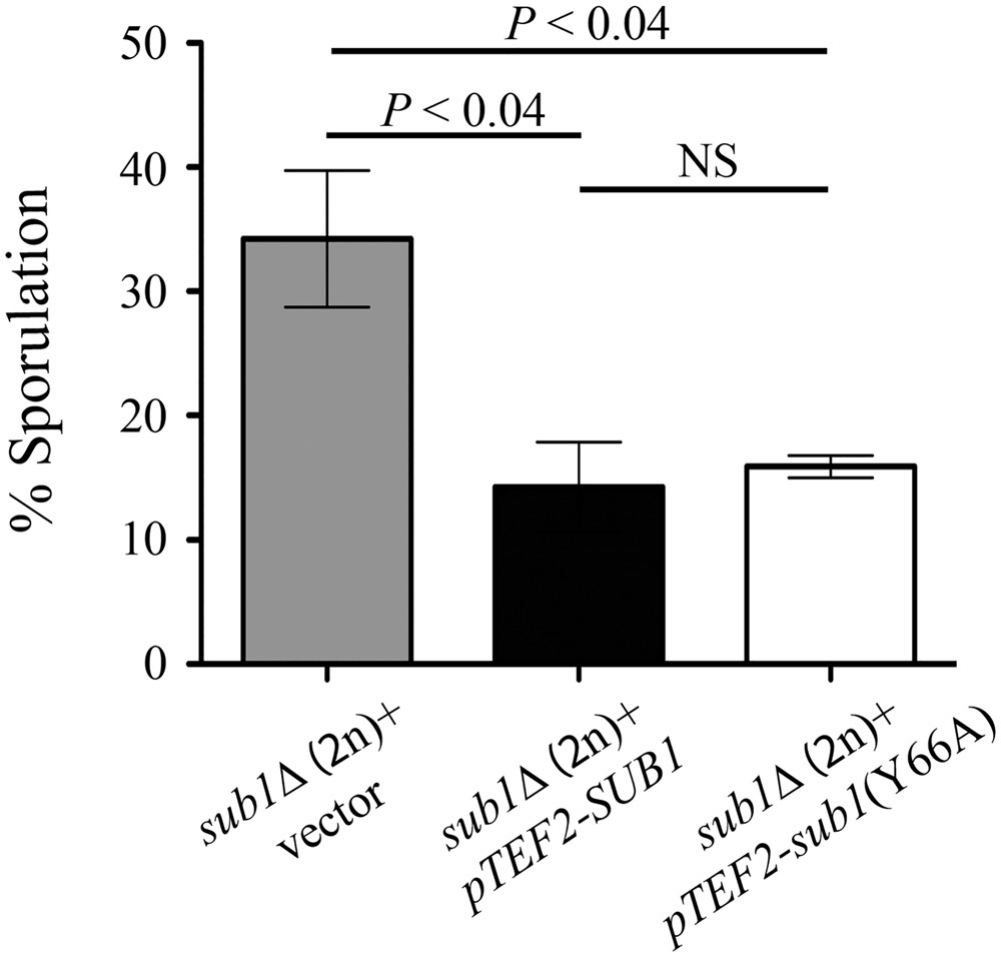
*sub1*(Y66A) missense mutant is competent for sporulation functions. Wild type *SUB1* or *sub1*(Y66A) mutant was over-expressed from *TEF2* promoter and *2μ* plasmid in S288c *sub1Δ/sub1Δ* strain. Sporulation was measured after 72 hours of transferring the cells to sporulation medium. Error bars represent standard deviation of three independent transformants for each strain (n = 2000) (NS, not significant).

Previously, it has been reported that Sub1 has a role in transcriptional repression of IMP dehydrogenase 2 (*IMD2)* [25]. Moreover, it is suggested that Sub1 and replication factor A (Rfa1) compete with each other for binding to ssDNA in transcription complexes. This conclusion was based on increased occupancy of Rfa1 at the promoter regions of several actively transcribed genes in haploid cells lacking Sub1 [5]. Interestingly the same study showed that *sub1*(Y66A) missense mutant showed increased *IMD2* gene expression with occupancy of Rfa1 at active promoters in the *sub1*(Y66A) missense mutant being similar to that in *sub1Δ* strain [5]. Because *sub1*(Y66A) missense mutant displayed phenotypes similar to the loss of function allele, in the haploid cells, we investigated the role of *sub1*(Y66A) mutant in sporulation response of diploid cells subjected to severe starvation. Towards this aim, we assessed sporulation efficiency of *sub1Δ/sub1Δ* S288c cells transformed with plasmid over-expressing either *sub1*(Y66A) mutant allele or the wild-type *SUB1*. Expression of the full length Sub1 protein reduced the sporulation efficiency to near S288c wild-type levels (Fig 2). Interestingly, the over-expression of *sub1*(Y66A) mutant in strain lacking endogenous Sub1 also showed a reduction in sporulation efficiency to levels achieved by the plasmid expressed wild type Sub1. This result suggests that the *sub1*(Y66A) missense mutant is competent for the sporulation functions of Sub1. To ascertain comparable protein levels, Western blot analysis was performed on protein lysates from *sub1Δ/sub1Δ* cells expressing GFP fusions with either wild-type *SUB1* or *sub1*(Y66A) missense mutant (S2A Fig). As we detect equivalent levels of Sub1(Y66A) and wild-type Sub1 proteins, we conclude that the missense mutation does not alter protein stability. Genome-wide immunolocalization study of yeast proteins in haploid log-phase cells reported Sub1 to be a nuclear protein [26]. We validated the nuclear localization of wild-type GFP-Sub1 and found that in haploid log-phase cells the GFP-Sub1(Y66A) mutant protein is also nuclear localized (S2B Fig). While functional for repression of sporulation in diploid cells, the *sub1*(Y66A) mutant does not repress *IMD2* gene expression, as the transcript levels of *IMD2* gene in *sub1*(Y66A) mutant were comparable to *sub1Δ* deletion strain (S2C Fig), in accord with a previous report of its effects on *IMD2* in haploid vegetative cells [5]. Taken together, our result indicates that *sub1*(Y66A) is competent for sporulation functions.

### Sub1 is repressed during sporulation

**Fig 3:**
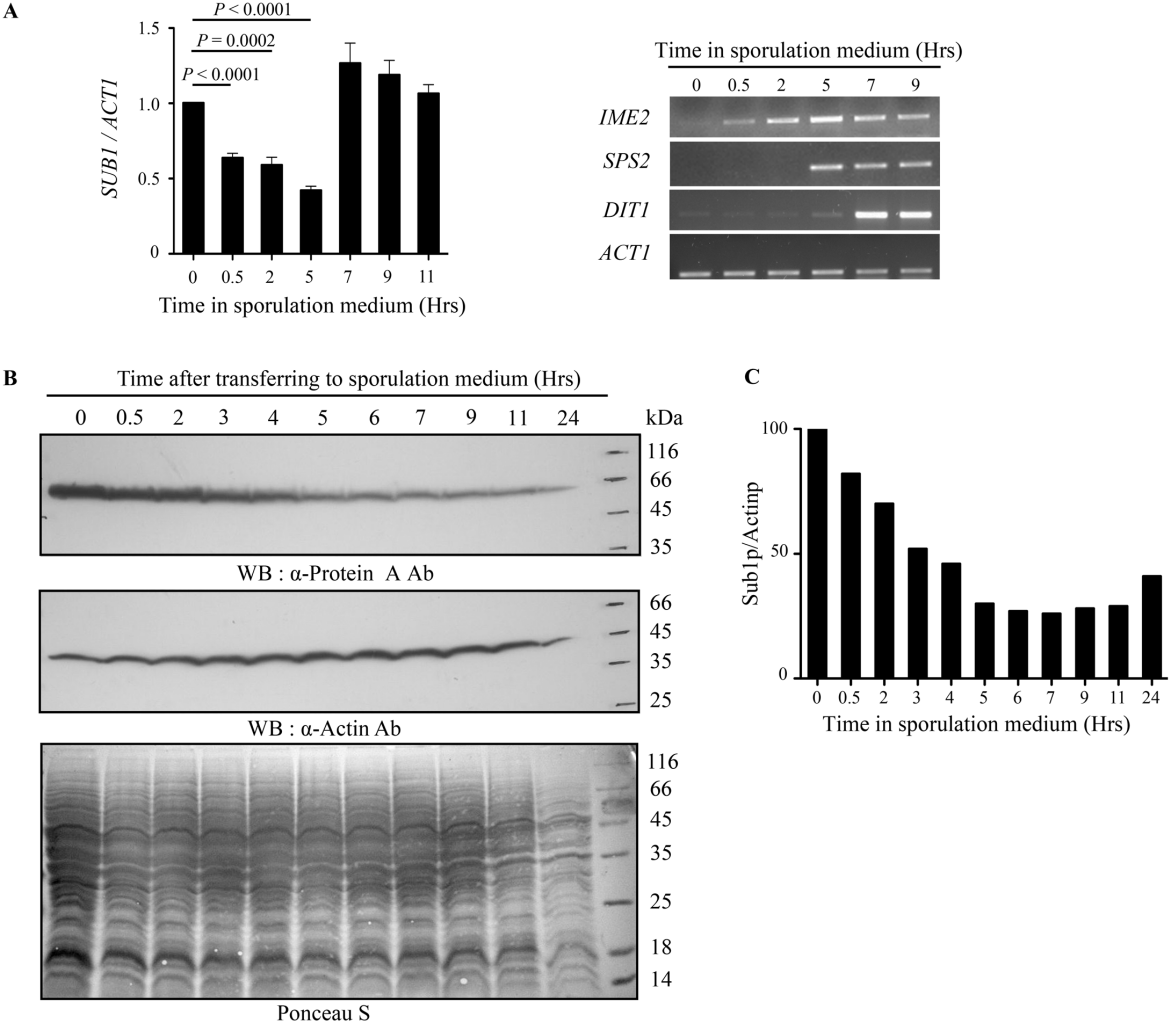
Sub1 decreases during sporulation. (A) WT SK1 cells were synchronized in YPA medium and then transferred to sporulation medium. Cells were collected at indicated time points. Normalization was done with *ACT1* levels. qRT-PCR analysis for *SUB1* transcript (left panel). Semi qRT-PCR analysis for early (*IME2*), middle (*SPS2*) and late (*DIT1*) sporulation-specific genes (right panel). (B) Western blot analysis of endogenously TAP tagged Sub1 protein for indicated time points. Sub1 protein is highest at the time of shifting the cells to sporulation medium and subsequently decreases over the sporulation time-course. Actin was used as a loading control. (C) Quantitation of Sub1 protein levels shown in (B).

Sporulation requires a regulated temporal expression of genes that facilitate meiosis and spore formation. Based on temporal expression patterns, sporulation genes are classified as early, middle, mid-late and late genes [15,16]. To determine Sub1 expression pattern during the sporulation cascade, we determined its transcript and protein levels. These experiments were performed in the SK1 strain as a very efficient and synchronous passage through sporulation is necessary to examine the temporally ordered sporulation gene expression which are characteristics seen in this strain. SK1 cells were first synchronised by growth in YPA medium and then transfer to sporulation medium. Culture aliquots were collected at different time points during sporulation for RNA isolation and expression studies. The *SUB1* transcript levels and transcript abundance for several other well-studied sporulation specific genes were measured by quantitative and semi-quantitative RT-PCR analysis respectively. *SUB1* transcript levels decreased after 30 minutes of transfer to sporulation medium and remained low for the subsequent 5 hours. The transcript levels increased again at 7th hour and remained high in the later time course of sporulation (Fig 3A left panel). This is in agreement with the previously reported high-density tiling microarray data analysed in SK1 strain during sporulation [24]. The window wherein *SUB1* transcripts were at the lowest, is correlated with the induction of expression of early and middle sporulation genes (Fig 3A right panel).

Consistent with reduced *SUB1* transcript levels during sporulation, our Western blot analysis revealed that Sub1 protein levels too decreased gradually on shift to sporulation medium (Fig 3B). Quantitation of Sub1 protein levels showed that by 3 hours, cells have about 50% of Sub1 as compared to the levels before transfer to starvation conditions (Fig 3C). This decrease in Sub1 protein levels during sporulation correlates with the onset of meiosis. Moreover, at later time points (7 hours post-induction and thereafter), Sub1 protein remained low. Together, our data shows that both *SUB1* transcript and protein levels reduces on transfer of cells to sporulation medium. However, we see a distinct pattern for *SUB1* transcript and protein levels during the later time-points of sporulation, which led us to speculate the possibility of post-transcriptional regulation of Sub1 transcript.

### Middle sporulation genes are the downstream targets of Sub1

**Fig 4:**
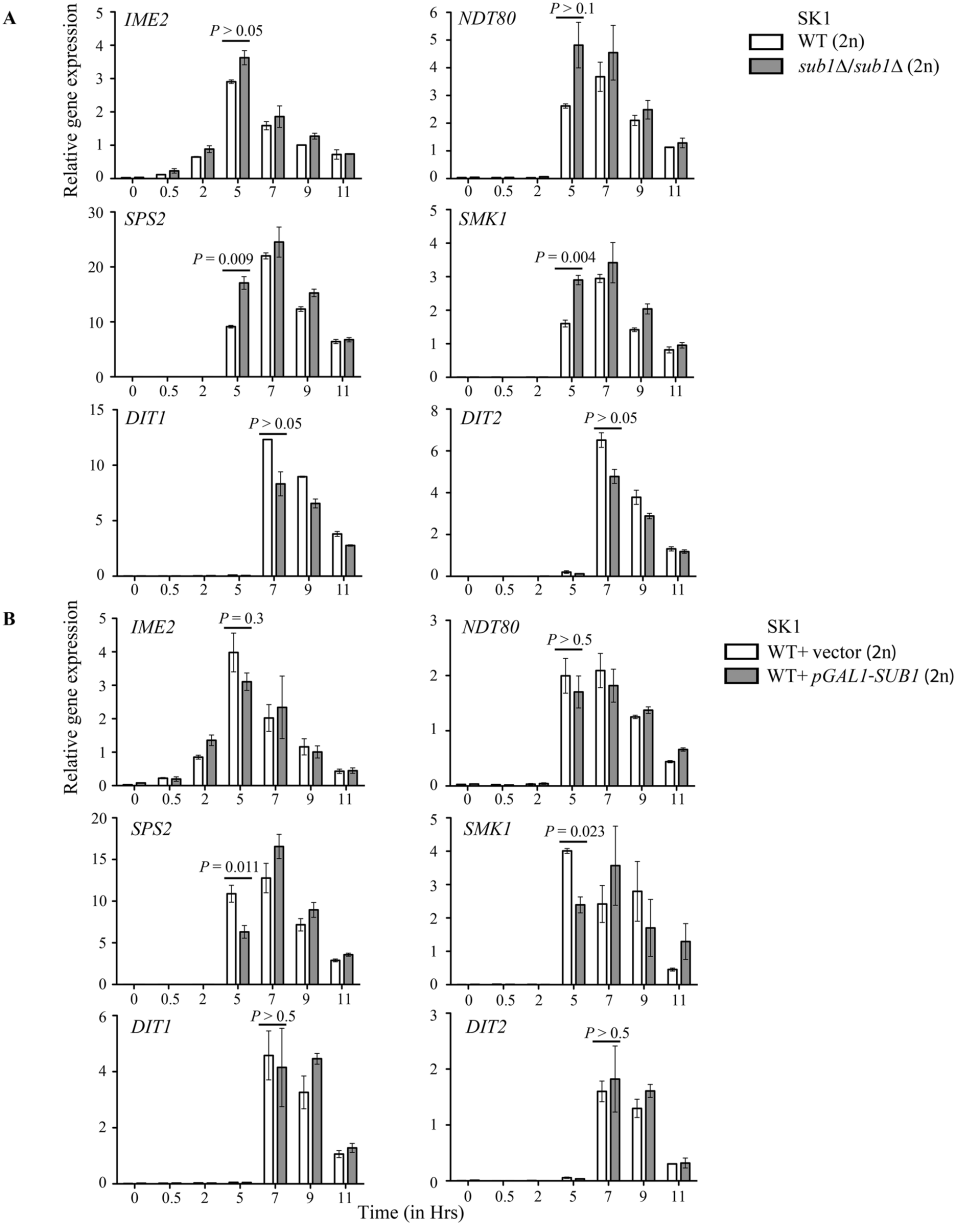
*SUB1* deletion results in increased expression of middle sporulation genes. qRT-PCR analysis for expression of early (*IME2*), early-middle (*NDT80*), middle (*SPS2*, *SMK1*) and mid-late (*DIT1*, *DIT2*) sporulation specific genes. Fold change was calculated using *ACT1* as control. (A) Cells were harvested at indicated time points from SK1 WT and *sub1Δ* cells. (B) Overexpression of *SUB1* results in decrease in expression of middle sporulation genes. Cells were harvested at indicated time points from SK1 WT and WT overexpressing *SUB1*.

Because Sub1 levels show a significant reduction correlating with progression of meiosis, we investigated its likely downstream effects on sporulation gene expression. We generated homozygous diploid *sub1Δ* alleles in the SK1 genetic background, so as to achieve synchronous and high sporulation. The wild-type and *sub1Δ/sub1Δ* SK1 diploids were allowed to sporulate for 24 hours and the cells were collected at different time points during sporulation. Fluorescence microscopy of DAPI-stained cultures revealed that *sub1Δ/sub1Δ* diploid cells completed meiosis similar to wild-type, as observed by the presence of four distinct DAPI-stained foci (S3A Fig). Moreover, the sporulation efficiency and the germination ability of haploid spores from *sub1Δ/sub1Δ* diploids was comparable to characteristics of wild-type diploids (S3B and S3C Fig). By qRT-PCR analysis, we quantitated the expression levels of some genes chosen as representatives for each temporal class of sporulation genes. We compared the transcript abundance in wild-type and *sub1Δ/sub1Δ* deletion strains. The genes where transcript levels showed at least 2-fold change and statistical significance of P < 0.05 were considered affected. We observed that *sub1Δ/sub1Δ* cells have a significantly elevated transcript levels of middle sporulation genes (*SPS2* and *SMK1*), that followed normal induction kinetics, i.e. 5 hours post transfer to sporulation medium (Fig 4A). The early gene *IME2* and early-middle gene *NDT80* displayed a marginal, but statistically insignificant increase in their transcript levels in *sub1Δ/sub1Δ* cells as compared to the wild-type. Moreover, the expression levels or timing for mid-late sporulation genes (*DIT1*, *DIT2*) did not differ in *sub1Δ/sub1Δ* strain as compared to the wild-type.

In order to support these observations on altered expression of some class of sporulation genes, when cells lack Sub1, we carried out experiments with cells that over-express *SUB1* from the *GAL1* promoter by transforming the high copy plasmid. However, a point to note is that growth and media conditions that were adopted to achieve *SUB1* overexpression in vegetatively growing diploids prior to sporulation are distinctly different from the media used in the previous experiments. *SUB1* expression was induced in mitotically growing SK1 diploids cells transformed with *pGAL1-SUB1-6XHis-HA-Protein A* plasmid, by the addition of galactose to raffinose containing media for 6 hours, after which cells were transferred to sporulation medium (as described in materials and methods). The expression of sporulation specific genes was analyzed by qRT-PCR analysis. After 24 hours of transferring the cells to sporulation medium, no change in the sporulation efficiency was seen (S4A Fig); overexpression of plasmid expressed Sub1 tagged protein was evident on Western blot analysis (S4B Fig). Importantly, we see that over-expression of *SUB1* in wild type SK1 cells caused reduced expression of middle-sporulation genes (Fig 4B). However, the expression levels of early gene *IME2*, early-middle gene *NDT80* and mid-late genes *DIT1*, *DIT2* were not altered significantly. Taken together, these results indicate that in the sporulation process, Sub1 could modulate the expression of middle sporulation genes.

### Sub1 binds to the promoters of middle sporulation genes to regulate their expression

**Fig 5:**
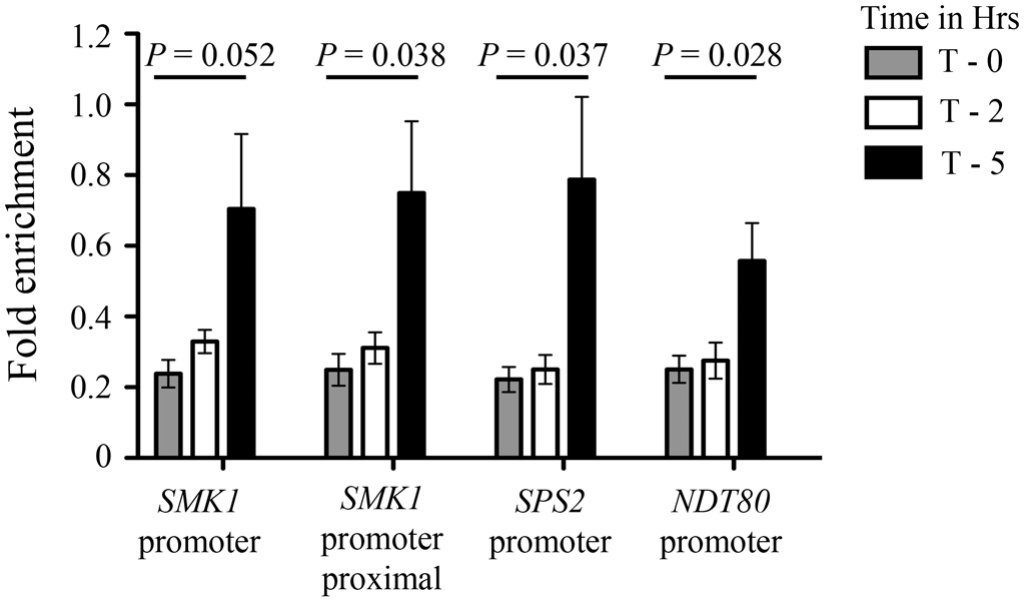
Sub1 recruitment at *SMK1* and *SPS2* middle gene loci. Chromatin immunoprecipitation analysis for recruitment of Sub1 at the promoters of early-middle (*NDT80*) and middle (*SMK1* and *SPS2*) sporulation genes at different time points (T—0, 2 and 5 hours post induction) during sporulation. Fold enrichment was calculated by normalization to *ACT1* promoter region.

In mitotically growing vegetative cells, Sub1 has been shown to associate with the promoter and coding regions of several constitutively—transcribed RNA Pol II and Pol III genes [3,4,6]. Moreover, Sub1 gets recruited to *IMD2* gene and thereby represses its expression [6,25]. These prior reports and our data highlighting the relationship between Sub1 and gene expression cascade during sporulation led us to hypothesize that Sub1 may be directly binding to the middle sporulation genes. To experimentally test this, we performed chromatin immunoprecipitation followed by quantitative PCR (ChIP-qPCR) in wild-type SK1 cells wherein the endogenous *SUB1* ORF was TAP tagged. These diploids were sporulated and chromatin was prepared from cells at 0, 2 and 5 hours after transfer to sporulation medium and subjected to immunoprecipitation. We detect significant enrichment of Sub1 at the promoter DNA sequence of *SMK1* and *SPS2* genes, notably at 5^th^ hour post transfer to sporulation medium (Fig 5). At the same time point the association of Sub1 was lowered at the *NDT80* promoter sequences as compared to the levels at *SMK1* and *SPS2* loci. This difference in Sub1 occupancy at *NDT80*, *SMK1* and *SPS2* loci temporally correlates with the increased transcript levels of *SMK1* and *SPS2* genes and the unaltered levels of *NDT80* transcripts in *sub1Δ/sub1Δ* SK1 cells. These data suggests that Sub1 shows a stronger association with middle sporulation genes and thereby regulates their expression levels.

### *SUB1* genetically interacts with *HOS2*

**Fig 6:**
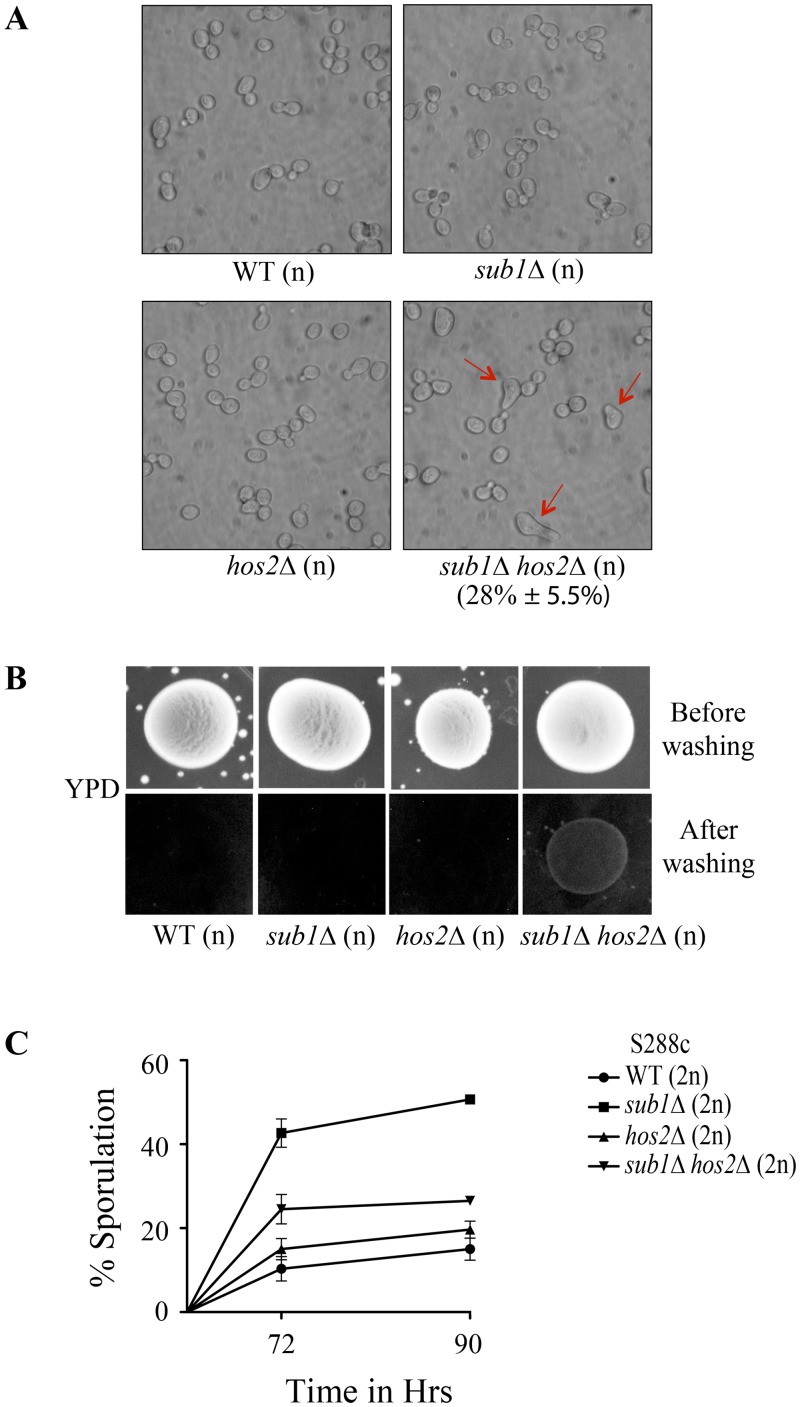
*SUB1* genetic interaction with *HOS2*. (A) Cell morphology of WT, *sub1Δ*, *hos2Δ* and *sub1Δ hos2Δ* (n) *MATa* cells photographed after 1 day of growth in liquid YPD medium. *sub1Δ hos2Δ* (n) double mutant cells show schmoo formation (indicated by arrows). (B) *sub1Δ hos2Δ* (n) double mutant strain shows agar invasion. WT, *sub1Δ*, *hos2Δ* and *sub1Δ hos2Δ* (n) strains were spotted on YPD medium and after 3 days of growth, plates were photographed, washed and re-photographed. (C) Sporulation was measured after 72 and 90 hours of transfer of cells from presporulation to sporulation medium. At least 600 cells were counted at each time point in two or three independent replicate experiments.

Genome-wide genetic interaction screens with vegetatively growing haploid cells, report Sub1 interactions with members of histone deacetylase complex, Set3C [27–30]. Here, we studied the genetic interaction of *SUB1* with *HOS2*, a subunit of Set3 complex. *SUB1* and *HOS2* are reported to show negative genetic interaction in haploid cells [27,29,30]. Moreover, *HOS2* is previously reported to be required for the response to secretory stress and the cells lacking Hos2 exhibit tunicamycin sensitivity [31]. We confirmed the sensitivity of *hos2Δ* strain to tunicamycin and further studied its genetic interaction with *SUB1* by examining its effects in *sub1Δ hos2Δ* strain. We observed a partial rescue of tunicamycin sensitivity in haploid *sub1Δ hos2Δ* cells (S5 Fig). Interestingly, a schmoo phenotype was noted in 28–30% of *sub1Δ hos2Δ* haploid cells which were grown in YPD medium, a phenotype not seen in either of the single mutants (Fig 6A). In *isw2Δ* cells, a mutant in a chromatin-remodelling complex subunit, autocrine activation of the pheromone response pathway results in schmoo formation and pheromone-induced agar invasion [32]. Thus, we investigated haploid *sub1Δ hos2Δ* double mutant for agar invasion and even on YPD medium *sub1Δ hos2Δ* cells display agar invasion (Fig 6B). Deletion of Hos2 is also reported to increase asci formation [33,34]. Genetic interactions between Sub1 and Hos2 during sporulation are not yet studied. Therefore, we generated homozygous a diploid *sub1Δ hos2Δ* strain and examined sporulation frequency of the double mutant as compared to the diploid with each of the single mutant loci and to wild-type diploids. *hos2Δ* diploids showed a marginal increase in sporulation when compared to the wild-type (Fig 6C). Moreover, we observed that sporulation efficiency of *sub1Δ hos2Δ* diploids was intermediate between those of the either single mutant strains. Taken together, these results suggest that *SUB1* and *HOS2* genetically interact during sporulation and may not be involved in a linear pathway.

### Human PC4 complements yeast *sub1Δ* phenotype

**Fig 7:**
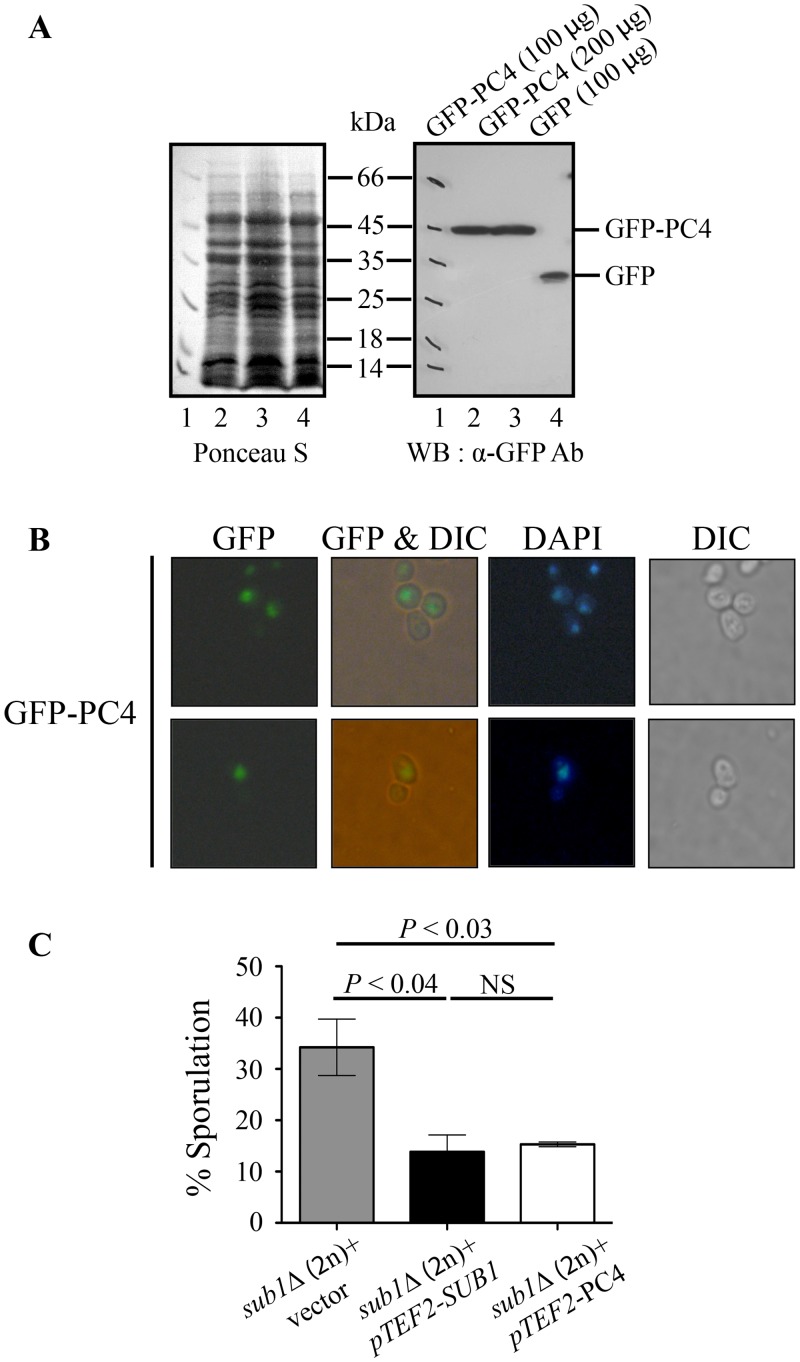
Human PC4 can complements the yeast *sub1Δ/sub1Δ* sporulation phenotype. (A) Protein lysates were prepared from strains expressing either GFP or GFP-PC4 protein and were resolved by SDS-PAGE. Western blotting was done with anti GFP antibodies. (B) Cells expressing GFP-PC4 protein were analyzed by fluorescence microscopy to detect GFP or DAPI signals. (C) Wild type yeast *SUB1* or Human PC4 was overexpressed from *TEF2* promoter and *2μ* plasmid in *sub1Δ/sub1Δ* strain. Sporulation was measured after 72 hours of transferring the cells to sporulation medium. Error bars represent standard deviation of three independent transformants for each strain (n = 1800) (NS, not significant).

*S*. *cerevisiae* Sub1 protein has a highly conserved N-terminal domain (1–107 aa), that has significant homology to even in its human counterpart PC4 [35] (S6 Fig). Besides this widely conserved domain is a C-terminal domain that is recognizable in its homologs from several other members of Saccharomycetaceae family like *Candida glabrata*, *Vanderwaltozyma polyspora*, *Zygosaccharomyces rouxii* and *Ashbya gossypii* (S6 Fig). The human PC4 (127 aa) and yeast Sub1 proteins (292 aa) share 48% identity and 70% similarity over the protein segment of 74 amino acids [1]. Therefore, we investigated whether human PC4 can complement the phenotypes of *S*. *cerevisiae sub1Δ* cells. We cloned the PC4 cDNA as N-terminal fusion with GFP in the yeast expression vector for expression from the *TEF2* promoter. PC4 protein levels were clearly detected by Western blot analysis (Fig 7A) and immunofluoresence studies showed as in human cells, PC4 is nuclear localized in yeast cells too (Fig 7B). Overexpression of PC4 in *sub1Δ/sub1Δ* cells showed a reduction in sporulation efficiency with the extent of reduction being similar to that seen on overexpression of yeast full length *SUB1*. These experiments demonstrate that the human PC4 protein functionally complements the *sub1Δ/sub1Δ* mutant phenotype (Fig 7C).

## Discussion

Sub1 has been implicated in various cellular processes—transcription [1–7,25,35], DNA repair [36], oxidative DNA damage [36] and osmotic stress response [4]. In this study, we have elucidated its regulatory functions during severe-starvation response of diploid yeast.

Diploids in the S288c strain background, with 12% ± 1.9% sporulation efficiency [37] are suited to identify genes that repress or reduce sporulation efficiency. Null mutants at such loci are expected to have increased sporulation efficiency as compared to isogenic wild-type S288c controls. A genome-wide screen for gene deletion mutants in S288c identified negative and positive regulators of sporulation [17], wherein Sub1 was classified as a negative regulator of sporulation. Such a genetic screen for negative regulators of sporulation is not feasible in wild-type SK1 strain, where sporulation efficiency is 92% ± 5.2% [37]. In S288c *sub1Δ/ sub1Δ* diploids, we note a nearly 5-fold increase in sporulation efficiency as compared with the isogenic wild-type diploids, 72 hours post-induction of sporulation and the efficiency (34% ± 2.5%) was largely similar to 40% sporulation efficiency as observed for deletion mutants of other negative regulators of sporulation [17]. In addition, we report that diploids with the *sub1*(Y66A) missense mutant have near wild-type sporulation efficiency. Moreover, we demonstrate strong Sub1 association at promoters of middle sporulation genes and show Sub1 can modulate their expression levels during sporulation. An earlier report showed that in haploid cells Sub1 chromatin association is not perturbed in the *sub1*(Y66A) mutant neither is the association of the single stranded DNA binding protein Rfa1 [5]. Our data show that in diploid cells this mutation does not impinge on starvation-inducted sporulation functions of Sub1.

In agreement with previously published genome-wide expression analysis [15,24], we report a rapid decrease in *SUB1* transcript levels on transfer of cells to sporulation medium which imposes severe starvation conditions. We show reduced transcript levels correlate with lowered Sub1 protein levels that persist at low level throughout the duration of sporulation. Under nutrient-rich conditions of vegetative growth, Ume6 in complex with Sin3/Rpd3 repressor [38–40] or Isw2 chromatin remodeling complex [41] acts as a transcriptional repressor of early sporulation genes. During starvation response, induction of Ime1 expression occurs, consequent to which Ume6 functions as an activator of early sporulation genes during meiotic growth [42,43]. Ume6 binds to the upstream response sequence (URS1) element present at its target genes [44,45]. Sub1 promoter element contains URS1 element positioned 674 bp upstream of transcriptional start site [46]. However, the significance of this Ume6 binding site in Sub1 promoter remains obscure. Further studies focused on understanding how Sub1 levels are controlled during sporulation will add to our findings and give better insights on its downstream effects.

By transcript analysis in *sub1Δ*/*sub1Δ* (2n) cells and by examining effects of *SUB1* over-expression in wild-type SK1 cells, we show that middle sporulation genes are its primary targets. Moreover, our temporal analysis of Sub1 occupancy at the promoter elements of middle genes establish first that Sub1 directly associates with chromatin at these loci. The time-kinetics of this recruitment coincide with time point when changes in their expression levels were noted when cells lacking Sub1 were put into the sporulation media.

Our genetic interaction studies were geared to understand how in S288c cells Sub1 acts to repress gene expression during sporulation and hence we studied its likely interactions with chromatin modifiers. Genome-wide genetic interaction screens performed with vegetatively growing haploid cells, report Sub1 interaction with several members of Set3C histone deacetylase complex, Set3C, including *SET3* [27,30], *HOS2* [27,29,30], *HST1* [28], *SNT1* [27,30] and *SIF2* [30,28]. Set3 complex (Set3C) comprises of seven subunits—Snt1, YIL112, Set3, Sif2, Hos2, Hst1, and Cpr1. Deletion of *SET3* or *HOS2* genes increases sporulation efficiency by affecting the early/middle sporulation genes [33,34]. In our experimental conditions we observed only a marginal increase in the sporulation efficiency in cells lacking Hos2 when compared to wild-type cells. A plausible reason for this could be the different sporulation conditions employed in previous study, where the S288C wild-type strain sporulates to a higher efficiency (~40%) [34]. However, S288C wild-type strain used in our studies showed 10–15% sporulation efficiency as is also reported in other studies [37,47]. We find genetic interactions between *SUB1* and *HOS2* in meiotic cells that suggests that a shared functional relationship. Hst1, a member of Set3C, is also known to be part of another repressor complex, Sum1. The latter complex is required to specifically repress middle sporulation genes by binding to middle sporulation element (MSEs) in their promoters. This binding and repression of gene expression occurs at the early and late phases of sporulation and also in vegetative cells [48]. The Ndt80 transcription factor also binds specifically to MSEs in the promoters of most of the middle genes but its binding leads to activation of their transcription [15,49]. Middle sporulation genes are reported to express in Ndt80-independent and dependent manner, that is, the first peak of expression arises from the removal of Sum1/Hst1 repression, which is Ndt80-independent [50]. However, a second later peak of expression for middle sporulation genes is Ndt80-dependent. In our expression data, Sub1 seems to affect only transcript levels during the first peak of middle gene expression (around 5 hours post-induction) while the expression levels during the second peak of expression (around 7 hours post-induction) remains unchanged. This is consistent with our data on *NDT80* expression, which show no significant affect on loss of Sub1 (Fig 4A and 4B). Therefore, we speculate that Sub1 affects the middle gene expression at the time of Sum1 complex removal and propose that this could be in an Ndt80-independent manner. The genome-wide genetic interaction data of Sub1 with Hst1, and with other subunits of Set3 complex, together suggest that Sub1 may function with Set3 or Sum1 repressor complexes.

Yeast Sub1 interacts with TFIIB basal transcription factor [1,2], while human PC4 requires interaction with TFIIA [51]. This difference in the preinitiation complex (PIC) interactions of PC4 vs. Sub1 has been attributed to the extended C-terminal region of Sub1, which is absent in PC4 [52]. In our analysis, human PC4 was found to complement the sporulation phenotype of *SUB1* deletion mutant, thus we speculate that some aspects of Sub1 function are conserved in yeast and humans, although the interacting factor in the basal transcription machinery likely differ.

## Supporting Information

S1 Fig Germination efficiency of *sub1Δ* mutant spores was similar to wild-type. Haploid spores obtained upon dissection of wild-type (2n) (top panel) and *sub1Δ/sub1Δ* (2n) (bottom panel) tetrads were allowed to germinate on YPD medium. 9 and 25 tetrads were dissected for wild-type and *sub1Δ/sub1Δ* (2n) strains respectively.
(TIF)

S2 Fig *sub1*(Y66A) missense mutant protein is stable, nuclear localized but does not affect *IMD2* gene expression. (A) The *sub1*(Y66A) missense allele does not affect protein stability. Protein lysates were prepared from strains expressing either GFP, GFP-Sub1 or GFP-Sub1Y66A protein and were resolved by SDS-PAGE. Western blotting was done with anti GFP antibodies. Asterik marked band is a cleaved product. (B) Localization of GFP-Sub1 and GFP-Sub1Y66A fusion proteins. Cells expressing different GFP-Sub1 fusion proteins or GFP alone as a control were analyzed by fluorescence microscopy to detect GFP or DAPI signals. Wild-type Sub1 and Sub1Y66A missense mutant localizes to the nucleus in yeast. (C) *sub1*(Y66A) mutant behaves like null strain with respect to *IMD2* expression. *IMD2* expression was measured in haploid *sub1Δ* cells expressing either wild type *SUB1* or *sub1*(Y66A) mutant. Normalization was done with *ACT1* levels. Error bars represent standard error of three independent RNA samples.
(TIF)

S3 Fig WT and *sub1Δ/sub1Δ* (2n) show comparable efficiency of meiosis, sporulation and germination of haploid spores. (A) WT and *sub1Δ/sub1Δ* (2n) cells were synchronized and transferred to sporulation medium. Cells were collected at different time points (0, 2, 5, 7, 9 and 11 hours) during sporulation. % veg (cells with one nucleus) and % tetrads (cells with three or four nuclei) were counted by fluorescence microscopy. (B) Sporulation was determined after transferring the cells to sporulation medium and % sporulation was plotted. Mature refractile asci were analyzed using DIC microscopy. At least 200 cells were counted for each sample. (C) Haploid spores obtained upon dissection of wild-type (2n) (top panel) and *sub1Δ/sub1Δ* (2n) (bottom panel) tetrads were allowed to germinate on YPD medium. 10 and 24 tetrads were dissected for wild-type and *sub1Δ/sub1Δ* (2n) strains respectively.
(TIF)

S4 Fig Overexpression of *SUB1* does not affect the sporulation efficiency in SK1 strain. (A) Wild type cells expressing either vector or *SUB1* were subjected to sporulation. Sporulation was measured at different time points after transfer of the cells to sporulation medium and % sporulation was plotted. Mature refractile asci were analyzed using DIC microscopy. At least 200 cells were counted for each sample. (B). Western blot analysis determines *SUB1* overexpression in WT SK1 strains. Protein lysates were prepared from strains at indicated time points after transferring them to sporulation medium. Western blotting was done with anti-His antibodies.
(TIF)

S5 Fig Deletion of *SUB1* in *hos2Δ* strain background partially rescues the tunicamycin sensitivity. WT (n), *sub1Δ* (n), *hos2Δ* (n) and *sub1Δ hos2Δ* (n) cells were spotted on synthetic complete medium and medium containing tunicamycin (1μg/ml). Plates were photographed after 2 days of growth at 28°C.
(TIF)

S6 Fig Multiple sequence alignment of *S*. *cerevisiae*, *Zygosaccharomyces rouxii*, *Vanderwaltozyma polyspora*, *Candida glabrata*, *Ashbya gossypii* and Human PC4 protein sequences. Alignments were determined using the CLUSTAL W2 computer alignment program. Identical amino acids are indicated by dark shades while similar amino acids in grey shades.
(TIF)

S1 Table Primers used in this study. (DOC)

## References

[1] Knaus R, Pollock R, Guarente L. Yeast *SUB1* is a suppressor of TFIIB mutations and has homology to the human co-activator PC4. EMBO J. 1996;15: 1933–1940. 8617240 PMC450112

[2] Henry NL, Bushnell DA, Kornberg RD. A yeast transcriptional stimulatory protein similar to human PC4. J Biol Chem. 1996;271: 21842–21847. 8702984 10.1074/jbc.271.36.21842

[3] Calvo O, Manley JL. The transcriptional coactivator PC4/Sub1 has multiple functions in RNA polymerase II transcription. EMBO J. 2005;24: 1009–1020. 10.1038/sj.emboj.7600575 15692559 PMC554125

[4] Rosonina E, Willis IM, Manley JL. Sub1 functions in osmoregulation and in transcription by both RNA polymerases II and III. Mol Cell Biol. 2009;29: 2308–2321. 10.1128/MCB.01841-08 19204085 PMC2663309

[5] Sikorski TW, Ficarro SB, Holik J, Kim T, Rando OJ, Marto JA, et al Sub1 and RPA associate with RNA polymerase II at different stages of transcription. Mol Cell. 2011;44: 397–409. 10.1016/j.molcel.2011.09.013 22055186 PMC3227220

[6] Garcia A, Collin A, Calvo O. Sub1 associates with Spt5 and influences RNA polymerase II transcription elongation rate. Mol Biol Cell. 2012;23: 4297–4312. 10.1091/mbc.E12-04-0331 22973055 PMC3484106

[7] Tavenet A, Suleau A, Dubreuil G, Ferrari R, Ducrot C, Michaut M, et al Genome-wide location analysis reveals a role for Sub1 in RNA polymerase III transcription. Proc Natl Acad Sci U S A. 2009;106: 14265–14270. 10.1073/pnas.0900162106 19706510 PMC2725013

[8] Koubova J, Guarente L. How does calorie restriction work? Genes Dev. 2003;17: 313–321. 10.1101/gad.1052903 12569120

[9] Lin SJ, Defossez PA, Guarente L. Requirement of NAD and *SIR2* for life-span extension by calorie restriction in *Saccharomyces cerevisiae* . Science. 2000;289: 2126–2128. 10.1126/science.289.5487.2126 11000115

[10] Mitchell AP. Control of meiotic gene expression in *Saccharomyces cerevisiae* . Microbiol Rev. 1994;58: 56–70. 8177171 10.1128/mr.58.1.56-70.1994 PMC372953

[11] Gimeno CJ, Ljungdahl PO, Styles CA, Fink GR. Unipolar cell divisions in the yeast *S*. *cerevisiae* lead to filamentous growth: Regulation by starvation and RAS. Cell. 1992;68: 1077–1090. 10.1016/0092-8674(92)90079-R 1547504

[12] Freese EB, Chu MI, Freese E. Initiation of yeast sporulation by partial carbon, nitrogen, or phosphate deprivation. J Bacteriol. 1982;149: 840–851. 7037742 10.1128/jb.149.3.840-851.1982 PMC216470

[13] Esposito MS, Esposito RE, Arnaud M, Halvorson HO. Acetate utilization and macromolecular synthesis during sporulation of yeast. J Bacteriol. 1969;100: 180–186. 5344095 10.1128/jb.100.1.180-186.1969 PMC315375

[14] Roman H, Phillips MM, Sands SM. Studies of polyploid *Saccharomyces*.I. Tetraploid segregation. Genetics. 1954;40: 546–561. 10.1093/genetics/40.4.546 PMC1209743 17247574

[15] Chu S, DeRisi J, Eisen M, Mulholland J, Botstein D, Brown PO, et al The transcriptional program of sporulation in budding yeast. Science. 1998;282: 699–705. 10.1126/science.282.5389.699 9784122

[16] Primig M, Williams RM, Winzeler EA, Tevzadze GG, Conway AR, Hwang SY, et al The core meiotic transcriptome in budding yeasts. Nat Genet. 2000;26: 415–423. 10.1038/82539 11101837

[17] Deutschbauer AM, Williams RM, Chu AM, Davis RW. Parallel phenotypic analysis of sporulation and postgermination growth in *Saccharomyces cerevisiae* . Proc Natl Acad Sci U S A. 2002;99: 15530–15535. 10.1073/pnas.202604399 12432101 PMC137751

[18] Goldstein AL, McCusker JH. Three new dominant drug resistance cassettes for gene disruption in *Saccharomyces cerevisiae* . Yeast. 1999;15: 1541–1553. 10514571 10.1002/(SICI)1097-0061(199910)15:14<1541::AID-YEA476>3.0.CO;2-K

[19] Janke C, Magiera MM, Rathfelder N, Taxis C, Reber S, Maekawa H, et al A versatile toolbox for PCR-based tagging of yeast genes: New fluorescent proteins, more markers and promoter substitution cassettes. Yeast. 2004;21: 947–962. 10.1002/yea.1142 15334558

[20] Mumberg D, Müller R, Funk M. Yeast vectors for the controlled expression of heterologous proteins in different genetic backgrounds. Gene. 1995;156: 119–122. 10.1016/0378-1119(95)00037-7 7737504

[21] Kassir Y, Simchen G. Monitoring meiosis and sporulation in *Saccharomyces cerevisiae* . Methods in Enzymology. 1991;194: 94–110. 10.1016/0076-6879(91)94009-2 2005827

[22] Lo H-C, Kunz RC, Chen X, Marullo A, Gygi SP, Hollingsworth NM. Cdc7-Dbf4 is a gene-specific regulator of meiotic transcription in yeast. Mol Cell Biol. 2012;32: 541–557. 10.1128/MCB.06032-11 22106412 PMC3255779

[23] Corbi D, Sunder S, Weinreich M, Skokotas A, Johnson ES, Winter E. Multisite phosphorylation of the Sum1 transcriptional repressor by S-phase kinases controls exit from meiotic prophase in yeast. Mol Cell Biol. 2014;34: 2249–63. 10.1128/MCB.01413-13 24710277 PMC4054303

[24] Kim Guisbert KS, Zhang Y, Flatow J, Hurtado S, Staley JP, Lin S, et al Meiosis-induced alterations in transcript architecture and noncoding RNA expression in *S*. *cerevisiae* . RNA. 2012;18: 1142–1153. 10.1261/rna.030510.111 22539527 PMC3358637

[25] Koyama H, Sumiya E, Nagata M, Ito T, Sekimizu K. Transcriptional repression of the *IMD2* gene mediated by the transcriptional co-activator Sub1. Genes to Cells. 2008;13: 1113–1126. 10.1111/j.1365-2443.2008.01229.x 18823333

[26] Huh W-K, Falvo J, Huh W-K, Falvo J V, Gerke L, Carroll A, et al Global analysis of protein localization in budding yeast. Nature. 2003;425: 686–91. 10.1038/nature02026 14562095

[27] Collins SR, Miller KM, Maas NL, Roguev A, Fillingham J, Chu CS, et al Functional dissection of protein complexes involved in yeast chromosome biology using a genetic interaction map. Nature. 2007;446: 806–810. 10.1038/nature05649 17314980

[28] Beltrao P, Trinidad JC, Fiedler D, Roguev A, Lim WA, Shokat KM, et al Evolution of phosphoregulation: Comparison of phosphorylation patterns across yeast species. PLoS Biol. 2009;7 10.1371/journal.pbio.1000134 PMC2691599 19547744

[29] Costanzo M, Baryshnikova A, Bellay J, Kim Y, Spear ED, Sevier CS, et al The genetic landscape of a cell. Science. 2010;327: 425–431. 10.1126/science.1180823 20093466 PMC5600254

[30] Zheng J, Benschop JJ, Shales M, Kemmeren P, Greenblatt J, Cagney G, et al Epistatic relationships reveal the functional organization of yeast transcription factors. Mol Syst Biol. 2010;6: 420 10.1038/msb.2010.77 20959818 PMC2990640

[31] Cohen TJ, Mallory MJ, Strich R, Yao TP. Hos2p/Set3p deacetylase complex signals secretory stress through the Mpk1p cell integrity pathway. Eukaryot Cell. 2008;7: 1191–1199. 10.1128/EC.00059-08 18487345 PMC2446675

[32] Frýdlová I, Basler M, Vašicová P, Malcová I, Hašek J. Special type of pheromone-induced invasive growth in *Saccharomyces cerevisiae* . Curr Genet. 2007;52: 87–95. 10.1007/s00294-007-0141-2 17639399

[33] Pim Pijnappel WWM, Schaft D, Roguev A, Shevchenko A, Tekotte H, Wilm M, et al The *S*. *cerevisiae SET3* complex includes two histone deacetylases, Hos2 and Hst1, and is a meiotic-specific repressor of the sporulation gene program. Genes Dev. 2001;15: 2991–3004. 10.1101/gad.207401 11711434 PMC312828

[34] Arévalo-Rodríguez M, Heitman J. Cyclophilin A is localized to the nucleus and controls meiosis in *Saccharomyces cerevisiae* . Eukaryot Cell. 2005;4: 17–29. 10.1128/EC.4.1.17-29.2005 15643056 PMC544151

[35] Calvo O, Manley JL. Evolutionarily conserved interaction between CstF-64 and PC4 links transcription, polyadenylation, and termination. Mol Cell. 2001;7: 1013–1023. 10.1016/S1097-2765(01)00236-2 11389848

[36] Wang J-Y, Sarker AH, Cooper PK, Volkert MR. The single-strand DNA binding activity of human PC4 prevents mutagenesis and killing by oxidative DNA damage. Mol Cell Biol. 2004;24: 6084–6093. 10.1128/MCB.24.13.6084-6093.2004 15199162 PMC480877

[37] Ben-Ari G, Zenvirth D, Sherman A, David L, Klutstein M, Lavi U, et al Four linked genes participate in controlling sporulation efficiency in budding yeast. PLoS Genet. 2006;2: 1815–1823. 10.1371/journal.pgen.0020195 PMC1636695 17112318

[38] Kurdistani SK, Robyr D, Tavazoie S, Grunstein M. Genome-wide binding map of the histone deacetylase Rpd3 in yeast. Nat Genet. 2002;31: 248–254. 10.1038/ng907 12089521

[39] Mallory MJ, Strich R. Ume1p represses meiotic gene transcription in *Saccharomyces cerevisiae* through interaction with the histone deacetylase Rpd3p. J Biol Chem. 2003;278: 44727–44734. 10.1074/jbc.M308632200 12954623

[40] Kadosh D, Struhl K. Repression by Ume6 involves recruitment of a complex containing Sin3 corepressor and Rpd3 histone deacetylase to target promoters. Cell. 1997;89: 365–71. 9150136 10.1016/s0092-8674(00)80217-2

[41] Goldmark JP, Fazzio TG, Estep PW, Church GM, Tsukiyama T. The Isw2 chromatin remodeling complex represses early meiotic genes upon recruitment by Ume6p. Cell. 2000;103: 423–433. 10.1016/S0092-8674(00)00134-3 11081629

[42] Rubin-Bejerano I, Mandel S, Robzyk K, Kassir Y. Induction of meiosis in *Saccharomyces cerevisiae* depends on conversion of the transcriptional represssor Ume6 to a positive regulator by its regulated association with the transcriptional activator Ime1. Mol Cell Biol. 1996;16: 2518–2526. 8628320 10.1128/mcb.16.5.2518 PMC231241

[43] Washburn BK, Esposito RE. Identification of the Sin3-binding site in Ume6 defines a two-step process for conversion of Ume6 from a transcriptional repressor to an activator in yeast. Mol Cell Biol. 2001;21: 2057–2069. 10.1128/MCB.21.6.2057-2069.2001 11238941 PMC86811

[44] Strich R, Surosky RT, Steber C, Dubois E, Messenguy F, Esposito RE. *UME6* is a key regulator of nitrogen repression and meiotic development. Genes Dev. 1994;8: 796–810. 10.1101/gad.8.7.796 7926768

[45] Anderson SF, Steber CM, Esposito RE, Coleman JE. *UME6*, a negative regulator of meiosis in *Saccharomyces cerevisiae*, contains a C-terminal Zn2Cys6 binuclear cluster that binds the URS1 DNA sequence in a zinc-dependent manner. Protein Sci. 1995;4: 1832–43. 10.1002/pro.5560040918 8528081 PMC2143208

[46] Harbison CT, Gordon DB, Lee TI, Rinaldi NJ, Macisaac KD, Danford TW, et al Scoring and clustering and conservation testing assignment of single motif to each regulator. Nature. 2004;431: 1–5. Published

[47] Deutschbauer AM, Davis RW. Quantitative trait loci mapped to single-nucleotide resolution in yeast. Nat Genet. 2005;37: 1333–1340. 10.1038/ng1674 16273108

[48] Lindgren A, Bungard D, Pierce M, Xie J, Vershon A, Winter E. The pachytene checkpoint in *Saccharomyces cerevisiae* requires the Sum1 transcriptional repressor. EMBO J. 2000;19: 6489–6497. 10.1093/emboj/19.23.6489 11101521 PMC305847

[49] Hepworth SR, Friesen H, Segall J. *NDT80* and the meiotic recombination checkpoint regulate expression of middle sporulation-specific genes in *Saccharomyces cerevisiae* . Mol Cell Biol. 1998;18: 5750–5761. 9742092 10.1128/mcb.18.10.5750 PMC109161

[50] Ahmed NT, Bungard D, Shin ME, Moore M, Winter E. The Ime2 protein kinase enhances the disassociation of the Sum1 repressor from middle meiotic promoters. Mol Cell Biol. 2009;29: 4352–4362. 10.1128/MCB.00305-09 19528232 PMC2725727

[51] Ge H, Roeder RG. Purification, cloning, and characterization of a human coactivator, PC4, that mediates transcriptional activation of class II genes. Cell. 1994;78: 513–523. 10.1016/0092-8674(94)90428-6 8062391

[52] Huang J, Zhao Y, Huang D, Liu H, Justin N, Zhao W, et al Structural features of the single-stranded DNA-binding protein MoSub1 from *Magnaporthe oryzae*. Acta Crystallogr D Biol Crystallogr. International Union of Crystallography; 2012;68: 1071–1076. 10.1107/S0907444912019932 22948907

