# Supplementary figures and images for: SUB1 Plays a Negative Role during Starvation Induced Sporulation Program in Saccharomyces cerevisiae
Source: PLoS One. 2015 Jul 6;10(7):e0132350. doi: 10.1371/journal.pone.0132350 (PMC4492983; doi:10.1371/journal.pone.0132350)

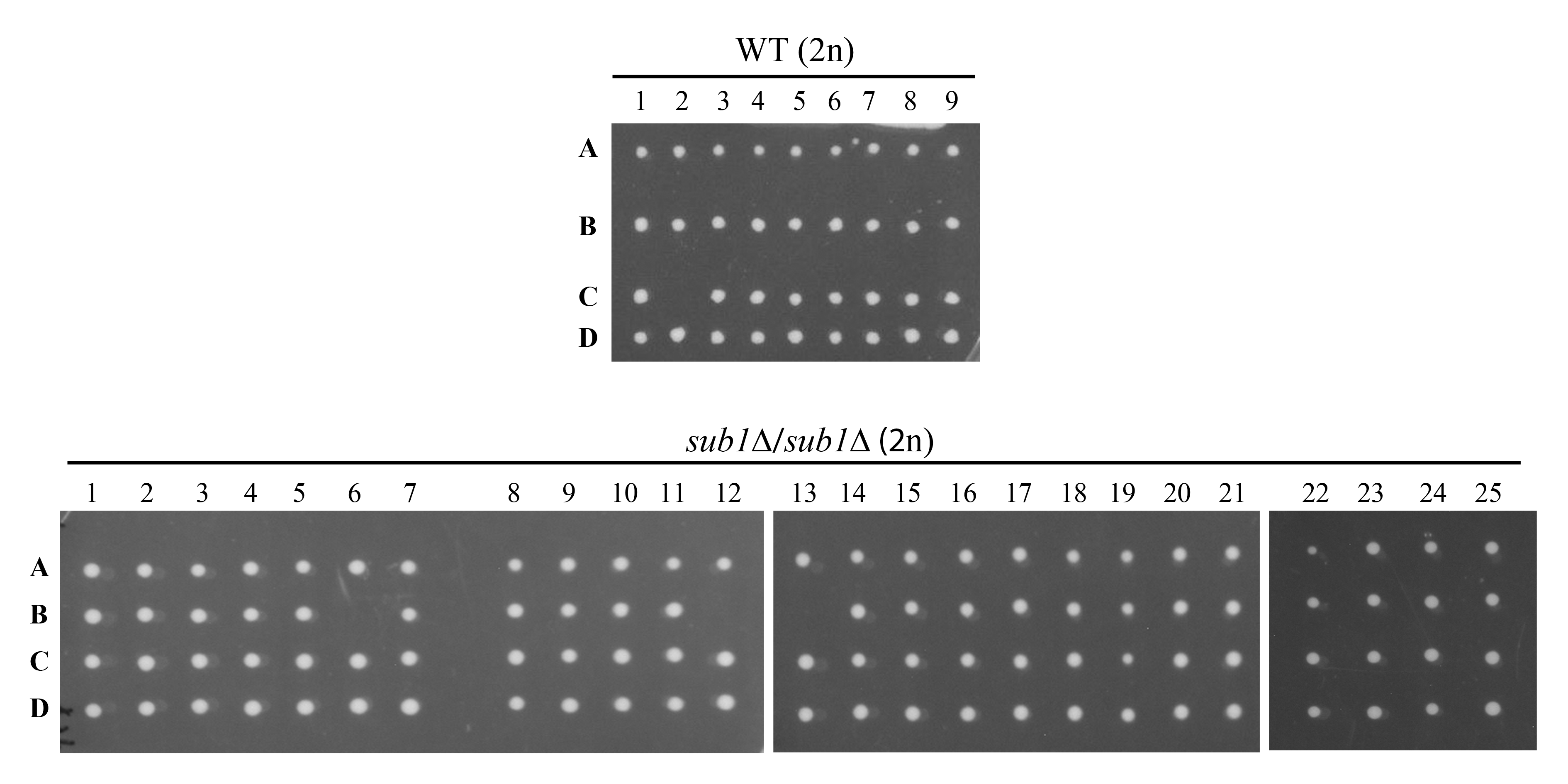

Supplement: S1 Fig — Haploid spores obtained upon dissection of wild-type (2n) (top panel) and sub1Δ/sub1Δ (2n) (bottom panel) tetrads were allowed to germinate on YPD medium. 9 and 25 tetrads were dissected for wild-type and sub1Δ/sub1Δ (2n) strains respectively. (TIF) [file pone.0132350.s001.tif]

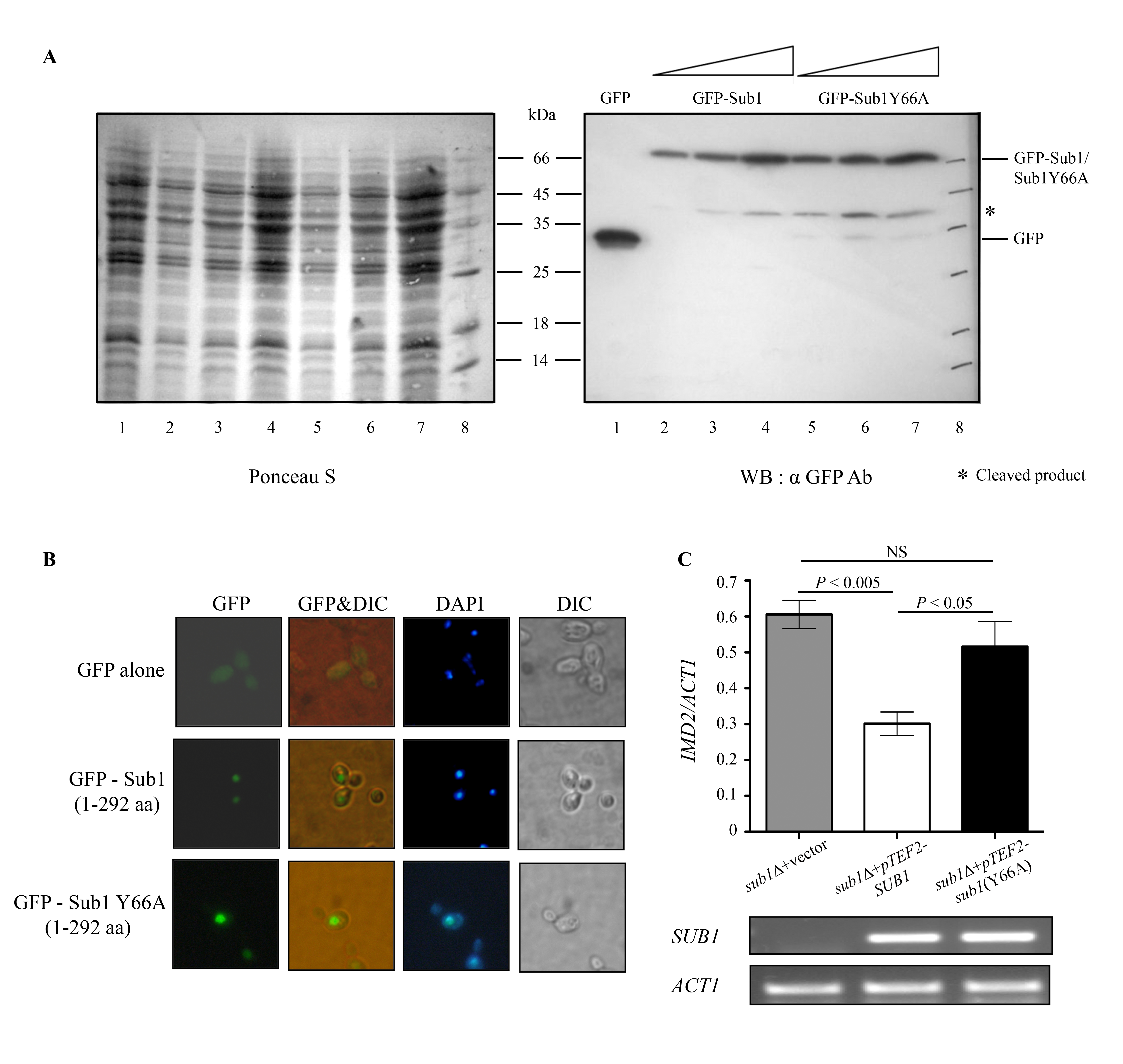

Supplement: S2 Fig — (A) The sub1(Y66A) missense allele does not affect protein stability. Protein lysates were prepared from strains expressing either GFP, GFP-Sub1 or GFP-Sub1Y66A protein and were resolved by SDS-PAGE. Western blotting was done with anti GFP antibodies. Asterik marked band is a cleaved product. (B) Localization of GFP-Sub1 and GFP-Sub1Y66A fusion proteins. Cells expressing different GFP-Sub1 fusion proteins or GFP alone as a control were analyzed by fluorescence microscopy to detect GFP or DAPI signals. Wild-type Sub1 and Sub1Y66A missense mutant localizes to the nucleus in yeast. (C) sub1(Y66A) mutant behaves like null strain with respect to IMD2 expression. IMD2 expression was measured in haploid sub1Δ cells expressing either wild type SUB1 or sub1(Y66A) mutant. Normalization was done with ACT1 levels. Error bars represent standard error of three independent RNA samples. (TIF) [file pone.0132350.s002.tif]

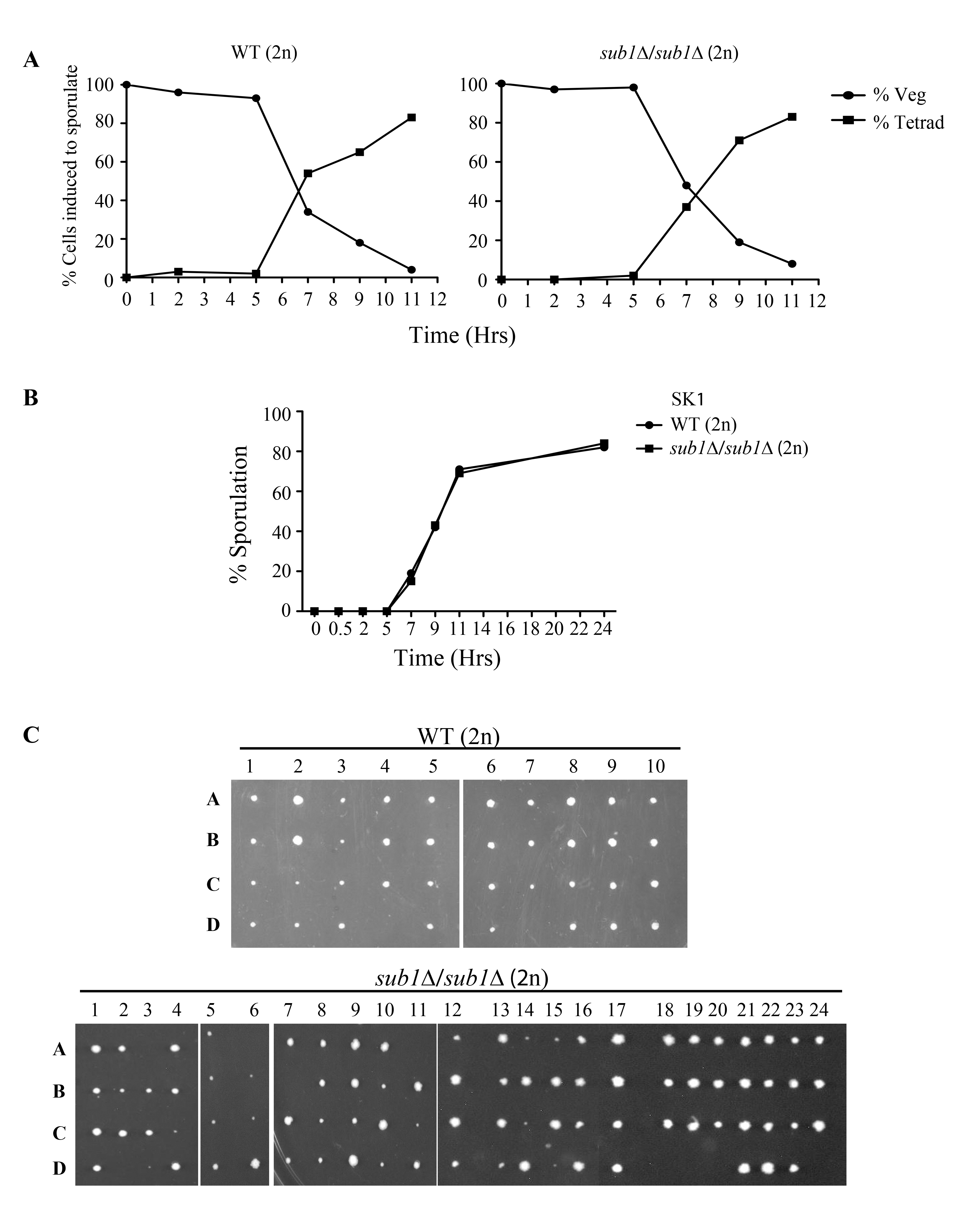

Supplement: S3 Fig — (A) WT and sub1Δ/sub1Δ (2n) cells were synchronized and transferred to sporulation medium. Cells were collected at different time points (0, 2, 5, 7, 9 and 11 hours) during sporulation. % veg (cells with one nucleus) and % tetrads (cells with three or four nuclei) were counted by fluorescence microscopy. (B) Sporulation was determined after transferring the cells to sporulation medium and % sporulation was plotted. Mature refractile asci were analyzed using DIC microscopy. At least 200 cells were counted for each sample. (C) Haploid spores obtained upon dissection of wild-type (2n) (top panel) and sub1Δ/sub1Δ (2n) (bottom panel) tetrads were allowed to germinate on YPD medium. 10 and 24 tetrads were dissected for wild-type and sub1Δ/sub1Δ (2n) strains respectively. (TIF) [file pone.0132350.s003.tif]

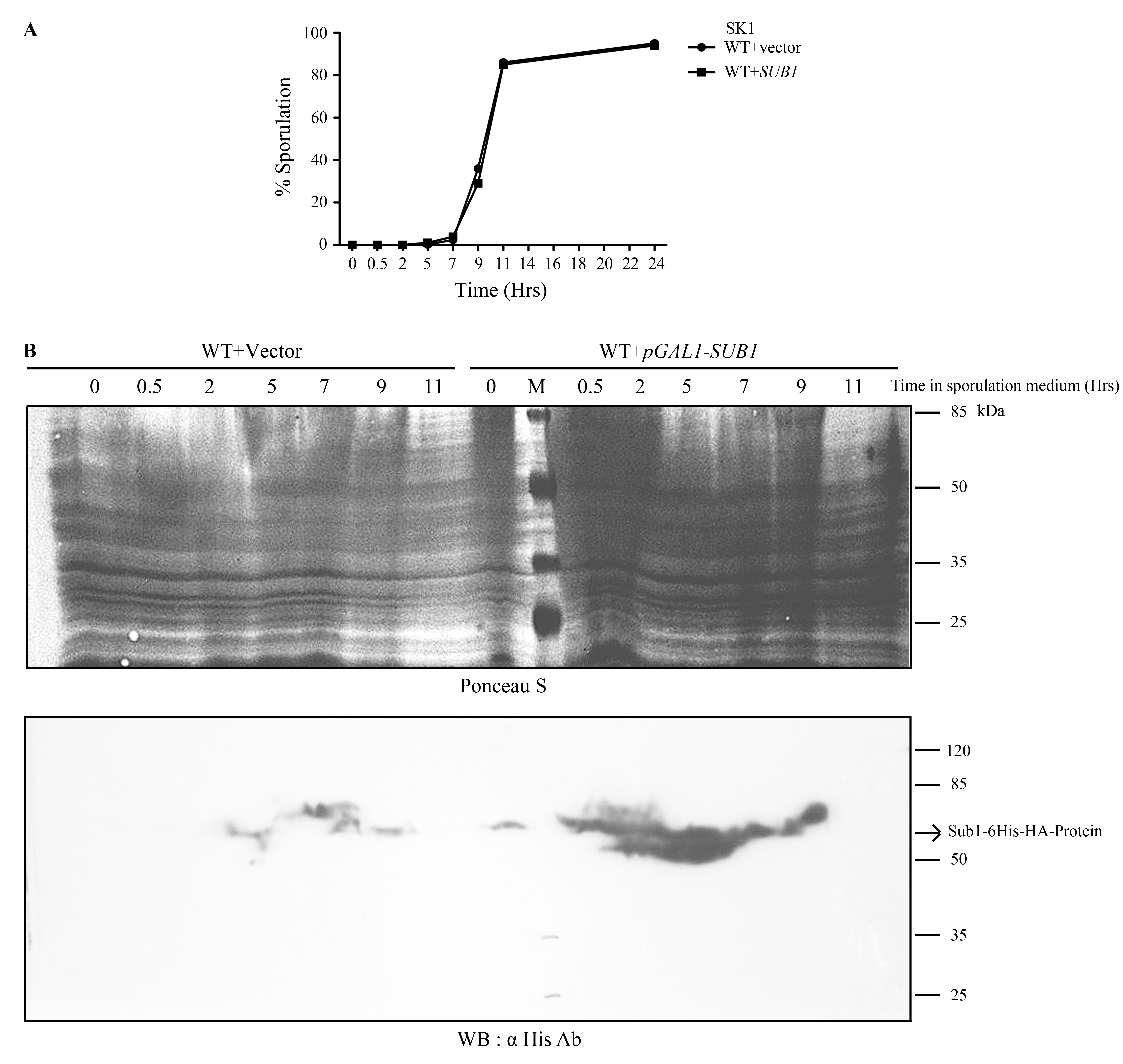

Supplement: S4 Fig — (A) Wild type cells expressing either vector or SUB1 were subjected to sporulation. Sporulation was measured at different time points after transfer of the cells to sporulation medium and % sporulation was plotted. Mature refractile asci were analyzed using DIC microscopy. At least 200 cells were counted for each sample. (B). Western blot analysis determines SUB1 overexpression in WT SK1 strains. Protein lysates were prepared from strains at indicated time points after transferring them to sporulation medium. Western blotting was done with anti-His antibodies. (TIF) [file pone.0132350.s004.tif]

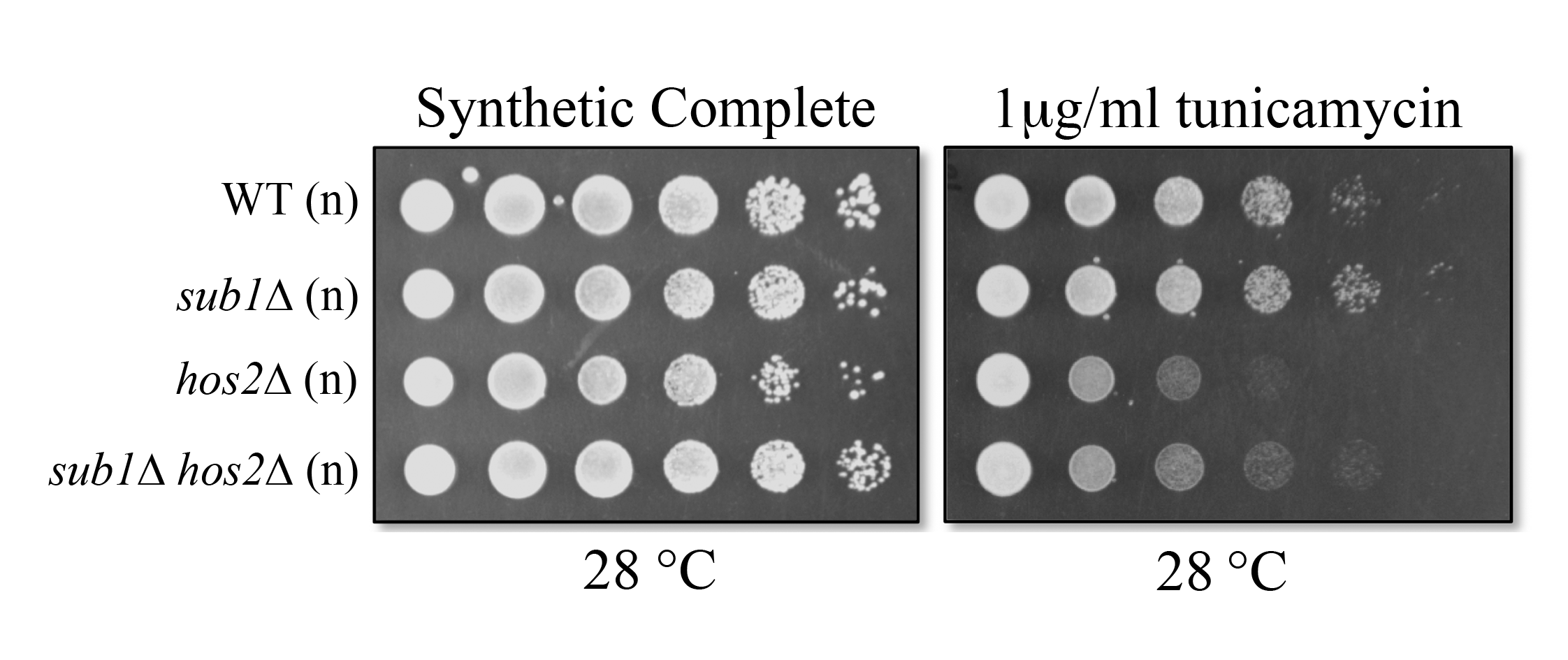

Supplement: S5 Fig — WT (n), sub1Δ (n), hos2Δ (n) and sub1Δ hos2Δ (n) cells were spotted on synthetic complete medium and medium containing tunicamycin (1μg/ml). Plates were photographed after 2 days of growth at 28°C. (TIF) [file pone.0132350.s005.tif]

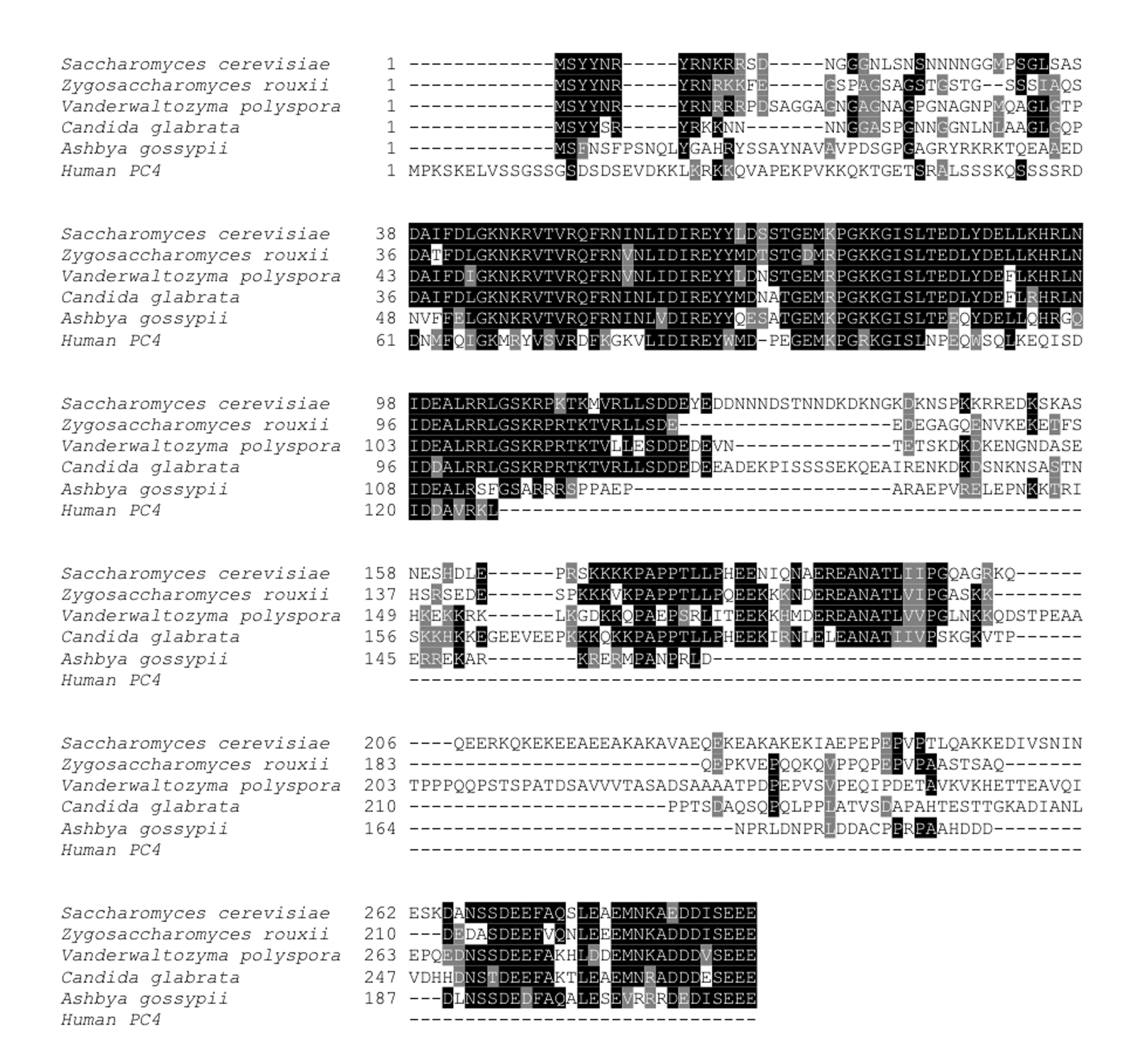

Supplement: S6 Fig — Alignments were determined using the CLUSTAL W2 computer alignment program. Identical amino acids are indicated by dark shades while similar amino acids in grey shades. (TIF) [file pone.0132350.s006.tif]
