# Supplementary material for: SUB1 Plays a Negative Role during Starvation Induced Sporulation Program in Saccharomyces cerevisiae
Source: PLoS One. 2015 Jul 6;10(7):e0132350. doi: 10.1371/journal.pone.0132350 (PMC4492983; doi:10.1371/journal.pone.0132350)
Supplement: S1 Table — (DOC) [file pone.0132350.s007.doc]

**S1 Table. Primers used in this study**

| **Primer Name** | **Sequence** |
| --- | --- |
| *SUB1*-TAP FP | 5’TCAAAGCTTGGAAGCTGAAATGAACAAGGCT GAAGACGACATAAGTGAAGAAGAACGTACGC TGCAGGTCGAC 3’ |
| *SUB1*-TAP RP | 5’AAGCTCGTTGGATGGAAGACGTTGACATAAG CAAGCTCAACTTCCAGGACTATTAATCGATGAATTCGAGCTCG 3’ |
| SK1 *SUB1* del FP | 5’TACACATCAATTTTTCGACATATATACAAAC ACAAGCGCTCGCCAGCTGAAGCTTCGTA 3’ |
| SK1 *SUB1* del RP | 5’TGGAAGACGTTGACATAAGCAAGCTCAACTT CCAGGACTAGAAGAGCGCCCAATACGC 3’ |
| *IME2* RT FP | 5’ TGTTTTGGGTGATGCCTCTTTA 3’ |
| *IME2* RT RP | 5’GGCTCGAGTTAGAAGGTTACATTTCCAAAATG 3’ |
| *NDT80* RT FP | 5’ CAGCTACGGAAGAGGATGTG 3’ |
| *NDT80* RT RP | 5’ GTAGAGTACCGCTTGCAATC 3’ |
| *SPS2* RT FP | 5’ GCAGGAAACTGACAATGATAATGG 3’ |
| *SPS2* RT RP | 5’ CGAGAACAATACCGTGAAAACTG 3’ |
| *SMK1* RT FP | 5’ AAACGAGGTGCGGAAGCCAG 3’ |
| *SMK1* RT RP | 5’ TAAAGACGAGGAGGACAAATCG 3’ |
| *DIT1* RT FP | 5’ AATTGTGTCGTGAAGGTTGAG 3’ |
| *DIT1* RT RP | 5' CTTGATAACGAAATGTCCTCC 3’ |
| *DIT2* RT FP | 5’ AAGAGCATGCCTAGGGGAAAA 3’ |
| *DIT2* RT RP | 5’ TAAATTAAGAGGGCAAAGAGGC 3’ |
| *ACT1* RT FP | 5’ CACCATGTTCCCAGGTATTGC 3’ |
| *ACT1* RT RP | 5’ TTGGAAGGTAGTCAAAGAAGCC 3’ |
| *SUB1* RT FP | 5’ TGAACCTGTACCCACACTTCAA 3’ |
| *SUB1* RT RP | 5’GGCTCGAGTTATTCTTCTTCACTTATGTCGT3’ |
| *SMK1* Promoter FP | 5’ GTTTGTTTGCCCACCGCTAA 3’ |
| *SMK1* Promoter RP | 5’ CAAGTGTCACAAATTAGTGGC 3’ |
| *SMK1* Promoter proximal FP | 5’ ACAGTATTTAACGTCGTGCGA 3’ |
| *SMK1* Promoter proximal RP | 5’ TGTTGAGGAGCACCGAGGTT 3’ |
| *SPS2* Promoter FP | 5’ TATTTACCTGCTTATTTCTCCC 3’ |
| *SPS2* Promoter RP | 5’ CTTTTATAGTCTAAGTAATGCCAC 3’ |
| *NDT80* Promoter FP | 5’ GCCGTATGACACAAAAGAGTA 3’ |
| *NDT80* Promoter RP | 5’ GGTGACACAAAATGGAGGGC 3’ |
| *ACT1* Promoter FP | 5’ TCTTCTTTACCCGCCACGC 3’ |
| *ACT1* Promoter RP | 5’ GAGAGATTGGGAAGGAAAGG 3’ |
